# Poorer sleep impairs brain health at midlife

**DOI:** 10.1038/s41598-023-27913-9

**Published:** 2023-02-01

**Authors:** Tergel Namsrai, Ananthan Ambikairajah, Nicolas Cherbuin

**Affiliations:** 1 Centre for Research on Ageing, Health and Wellbeing, Australian National University, 54 Mills Road, Canberra, ACT 2601 Australia; 2 Discipline of Psychology, Faculty of Health, University of Canberra, Canberra, ACT 2617 Australia

**Keywords:** Dementia, Alzheimer's disease, Sleep disorders

## Abstract

Sleep is an emerging risk factor for dementia but its association with brain health remains unclear. This study included UK Biobank (n = 29,545; mean age = 54.65) participants at imaging visit with sleep measures and brain scans, and a subset (n = 14,206) with cognitive measures. Multiple linear regression analyses were conducted to study the associations between sleep and brain health. Every additional hour of sleep above 7 h/day was associated with 0.10–0.25% lower brain volumes. In contrast, a negative non-linear association was observed between sleep duration, grey matter, and hippocampal volume. Both longer (> 9 h/day) and shorter sleep (< 6 h/day) durations were associated with lower brain volumes and cognitive measures (memory, reaction time, fluid intelligence). Additionally, daytime dozing was associated with lower brain volumes (grey matter and left hippocampus volume) and lower cognitive measures (reaction time and fluid intelligence). Poor sleep (< 6 h/day, > 9 h/day, daytime dozing) at midlife was associated with lower brain health. Sleep may be an important target to improve brain health into old age and delay the onset of dementia.

## Introduction

Dementia is a major burden of disease with rising social and economic costs. Globally, 28.8 million disability-associated life years are attributable to dementia^1^. It is the fifth leading cause of death costing $818 billion annually worldwide^1^. Additionally, the prevalence of dementia has more than doubled in the last 30 years^2^ and is projected to increase from 50 million in 2016 to 150 million by 2050^3^.

Since there is no cure for dementia and its prevalence is increasing due to an ageing population, identifying risk factors, and developing risk-reduction interventions is currently the most promising option. Twin studies suggest 58–79% of dementia cases are attributable to genetics^4^. This implies that 21–42% of dementia cases are attributable to non-genetic factors that are modifiable. Research has investigated the contribution of several modifiable risk factors for dementia and found that the main known risk factors explain only 35% to 40% of dementia cases^4,5^. These include smoking, alcohol misuse, high blood pressure, diabetes, mid-life obesity, physical inactivity, depression, lower cognitive activity, hearing loss, air pollution and head injury^4^. Thus, a substantial proportion of potentially modifiable risk factors has not been identified.

An emerging modifiable risk factor for dementia is sleep. The evolutionary origins and the role of sleep in maintaining healthy cerebral function are not well-understood. However, sleep is known to regulate brain cellular homeostasis by clearing debris, misfolded proteins, and other toxic byproducts^5^ from the interstitial fluid via a paravascular pathway known as the glymphatic system^6^. In humans, poor sleep is associated with increased dementia pathology including amyloid-β deposition^7,8^, taupathy^9^, and α-synuclein load^10^. Despite the expanding body of literature suggesting an association between sleep and dementia, we have a limited understanding of the way in which sleep characteristics affect brain health and cognition at midlife.

Pathological changes linked with the development of dementia are known to occur decades before its clinical onset^11^. Since at midlife major pathological changes are more likely to be reversible, implementation of interventions aimed at reducing modifiable risk factors exposure during or before this time point in life could have a greater effect than implementing them later on, as irreversible pathological changes are less likely to have already occurred. Thus, investigating the role of sleep as a possible early modifiable risk factor for dementia that may occur at midlife, or before, is of particular interest.

Moreover, whether possible effects of sleep on cognitive functions are mediated by brain health is unclear. Thus, in this study we used brain volumes as markers of brain health as smaller brain volumes have been shown to be indicators of neuronal and glial cells loss, atrophy of dendritic trees, and cellular shrinkage^12^. Additionally, both brain volumes and cognition have been shown to vary by age and sex^12,13^. Women have on average better memory scores but faster cognitive decline compared to men^13^. In contrast, men tend to have larger brain volumes but experience steeper decline in some regions including total brain volume, GM, and some WM (frontal, parietal) regions^12^. Therefore, another aim of this study was to investigate the associations between sleep characteristics, brain volumes and cognitive functions (as indexes of brain health), in middle-aged community-living individuals while considering the effects of age and sex. This will help clarify how age and sex (individually) modulate the associations between sleep characteristics and brain volumes, as well as cognitive functions. Moreover, as previous literature has reported lower brain volumes in older people sleeping more than 7 hs per day^14,15^, and not only in the context of short sleep durations, this study aimed to investigate the possibility of non-linear association between sleep characteristics and brain health in middle-aged adults. Finally, we also aimed to investigate whether poorer brain health indexed by smaller brain volumes, might explain at least in part the associations between sleep and cognitive functions.

## Results

Participants characteristics are presented in Supplementary Table 1. The average age of the study population was 54.65 years (95% CI = 54.5 to 54.7) at baseline and 63.00 years (95% CI = 62.9 to 63.0) at the imaging visit and included more males (55.42%, n = 13,765). On average, participants reported sleeping 7.16 h (95% CI = 7.15 to 7.17) at the baseline and 7.14 h (95% CI = 7.12 to 7.15) at the imaging visits. Of those 72.2% (baseline) and 77.7% (imaging) experienced insomnia symptom characterised by “difficulty falling asleep or waking up at night”, 36.4% (baseline) and 36.6% (imaging) reported snoring, 20.2% (baseline) and 23% (imaging) reported dozing. Males had significantly larger GM, WM, LHC and RHC volumes, and performed significantly better on the RT, NM, and FI tasks than females.

### Sleep measures, sex, and BMI

Associations between sleep and demographic measures are presented in Supplementary Table 2. For sleep, every 1-year increase in age above 55 was associated with 0.24 min (0.05%) shorter sleep duration at baseline and 0.18 min (0.04%) longer sleep duration at the imaging visits. Males slept 5.22 min (1.2%) and 4.5 min (1.1%) less than females at the baseline and the imaging visits. Moreover, every additional year in age above 55 was associated with 1.26 min (0.3%) longer sleep in males compared to females. Those classified as overweight had 1.9 min (0.4%) and those classified as obese had 5.6 min (1.3%) less sleep at the baseline than those with normal BMI. At the imaging visit, those with obesity had 0.66 min (0.16%) less sleep than those with normal BMI.

Association between sleep duration and other sleep variables (daytime dozing, chronotype, insomnia symptom, napping, difficulty getting up in the morning and snoring) are presented in Supplementary Table 3. Reporting napping, snoring, or difficulty getting up in the morning was associated with longer sleep duration while having insomnia symptom, daytime dozing and being an evening person was associated with significantly shorter sleep duration.

### Sleep characteristics and brain structures

Associations between sleep characteristics and brain volumes are summarized in Table 1 (refer to Supplementary Table 4 for full models). Every additional hour in sleep above 7 h/day at baseline was associated with 0.77 ml (0.1%, 95% CI = −1.12 to −0.41, β estimate: −0.012) lower GM, but not at the imaging visit. In contrast, every additional hour above 7 h/day was associated with 0.57 ml (0.1%, 95% CI = −0.93 to −0.22, β estimate: −0.009) and 0.51 ml (0.1%, 95% CI = −0.83 to −0.18; β estimate: −0.009) lower WM at the baseline and the imaging visit. Association between sleep duration at the imaging and GM and WM differed with age as shown in Supplementary Table 5. Every one-hour increase in sleep duration at the imaging visit in age above 55 was associated with 0.08 ml and 0.07 ml smaller GM and WM at the imaging visit (0.01% of GM and WM). Additionally, every additional hour in sleep duration was associated with 0.007 ml (0.25%, 95% CI = −0.01 to −0.003, β estimate: −0.02) and 0.007 ml (0.2%, 95% CI = −0.01 to −0.003, β estimate: −0.017) lower volume in both LHC and RHC at the imaging visit. Association between sleep duration at the imaging visit and hippocampal volumes differed with age and sex (Supplementary Table 5). Every additional hour in sleep duration at the baseline in those participants above 55 was associated with 0.001 ml smaller volume in RHC and 0.001 ml larger volume in LHC at the imaging visit (0.02% of LHC and RHC) compared to those below 55. Every additional hour in sleep duration at the baseline in males was associated with 0.01 ml lower volume in LHC and RHC at the imaging visit while it was associated with 0.01 ml lower volume in LHC at the imaging visit (0.2% of LHC and RHC). Based on the standardized β estimates, the effect of baseline sleep duration on WM was stronger than the effect on GM, LHC and RHC (Supplementary Table 6). When considering a categorical measure of sleep duration (< 6 h/day; > 9 h/day), a significant negative quadratic association for sleep duration was detected such that both low and high sleep durations were associated with lower GM volume (Table 1). However, only long sleep duration was associated with lower LHC and RHC volumes.

**Table 1:** Association between sleep characteristics and brain volumes (n = 29,545).

Independent variables			Dependent variables			
			GM (ml)	WM (ml)	LHC (ml)	RHC (ml)
Centred sleep duration^†^ (hours/day, 95% CI)	Baseline		−0.77* (−1.12, −0.41)	−0.57* (−0.93, −0.22)	−0.01* (−0.01, −0.01)	−0.01* (−0.01, −0.01)
	Imaging		−0.31(−0.63, 0.02)	−0.51* (−0.83, −0.18)	−0.007* (−0.01, −0.003)	−0.007* (−0.01, −0.003)
Adjusted R^2^	Baseline		0.765	0.742	0.365	0.354
	Imaging		0.765	0.744	0.369	0.357
Categorical sleep duration^‡^ (95% CI)	Baseline	Short	−1.76 (−3.74, 0.22)	1.29 (−0.70, 3.27)	−0.01 (−0.01, 0.03)	−0.01 (−0.03, 0.02)
		Long	−4.13* (−6.94, −1.31)	−1.21 (−4.04, 1.61)	−0.02 (−0.05, 0.02)	−0.02 (−0.06, 0.01)
	Imaging	Short	−3.74* (−5.33, −2.14)	0.22 (−1.38, 1.82)	−0.01 (−0.03, 0.01)	−0.02 (−0.03, 0.004)
		Long	−4.98* (−7.81, −1.64)	−2.53 (−5.38, 0.32)	−0.05* (−0.02, −0.02)	−0.06* (−0.10, −0.03)
Adjusted R^2^	Baseline		0.764	0.741	0.365	0.354
	Imaging		0.765	0.743	0.369	0.359
Quadratic sleep duration (95% CI)	Baseline		−0.571* (−0.82, −0.32)	−0.04 (−0.29, 0.21)	−0.002 (−0.005, 0.001)	−0.005* (−0.01, −0.002)
	Imaging		−0.735* (−0.94, −0.53)	−0.092 (−0.29, 0.11)	−0.004* (−0.01, −0.002)	−0.005* (−0.01, −0.002)
Adjusted R^2^	Baseline		0.765	0.742	0.365	0.354
	Imaging		0.765	0.744	0.0.369	0.357
Daytime dozing (yes/no, 95% CI)	Baseline		−1.80* (−2.63, −0.98)	−0.96 (−1.79, −0.14)	−0.02* (−0.02, −0.01)	−0.01 (−0.02, −0.002)
	Imaging		−1.63* (−2.42, −0.84)	−1.18* (−1.97, −0.39)	−0.01* (−0.02, −0.004)	−0.01 (−0.02, −0.001)
Adjusted R^2^	Baseline		0.765	0.320	0.365	0.354
	Imaging		0.765	0.321	0.369	0.357
Snoring (yes/no, 95% CI)	Baseline		−0.15 (−0.87, 0.52)	0.04 (−0.60, 0.77)	0.00 (−0.01, 0.01)	0.00 (−0.01, 0.01)
	Imaging		0.51 (−0.20, 1.21)	0.20 (−0.50, 0.91)	0.02 (0.001, 0.02)	0.01 (0.001, 0.02)
Adjusted R^2^	Baseline		0.764	0.742	0.365	0.365
	Imaging		0.765	0.744	0.369	0.369
Insomnia symptom (yes/no, 95% CI)	Baseline		1.04* (0.30, 1.78)	1.41* (0.66, 2.16)	0.01 (0.00, 0.02)	0.01 (−0.004, 0.02)
	Imaging		0.40 (−0.40, 1.20)	0.83 (0.03, 1.63	0.01 (0.001, 0.02)	0.01* (0.004, 0.02)
Adjusted R^2^	Baseline		0.765	0.743	0.365	0.353
	Imaging		0.765	0.744	0.369	0.357

The estimates are controlled for the following covariates: age, sex, education, body mass index, smoking, alcohol, physical activity, hypertension, and diabetes.

*95% CI* 95% confidence interval.

*p-value < 0.0125; p-value is considered significant after Bonferroni correction.

^†^Sleep duration is centred on 7 h of sleep per day.

^‡^Normal sleep duration refers to 6–9 h of sleep per day; normal sleep duration is the reference group; short sleep duration refers to less than 6 h of sleep per day; long sleep duration refers to more than 9 h of sleep per day.

In addition, significant interactions between sex and sleep duration were detected for LHC and RHC at the baseline visit such that in females every additional hour in sleep duration was associated with larger volumes (0.01 ml) relative to males (Supplementary Table 5). For LHC only, interactions between sex and sleep duration were also found at the imaging visit such that in females every additional hour in sleep duration at the imaging visit was associated with smaller LHC (0.01 ml) compared to males. Additionally, an interaction between age and sleep duration was found for GM, WM and RHC at the imaging visit where in people over 55, every additional hour in sleep duration was associated with smaller volumes (GM = 0.58 ml vs 0.66 ml, WM = 0.49 ml vs 0.56 ml, RHC = 0.009 ml vs 0.01 ml).

Associations between daytime dozing, snoring, insomnia symptom, and brain volumes are presented in Table 1. Daytime dozing was associated with lower GM at the baseline (1.80 ml; 0.3%, 95% CI = −2.63 to 0.98, β estimate: −0.03) and the imaging (1.63 ml; 0.2%, 95% CI = −2.42 to 0.94; β estimate: −0.028) visits. In addition, daytime dozing was associated with lower LHC at the baseline (0.02 ml; 0.4%, 95% CI = −0.02 to −0.01; β estimate: −0.02) and the imaging (0.013 ml; 0.3%, 95% CI = −0.02 to −0.004, β estimate: −0.031) visits. Comparison of the standardized β estimates indicate that the effect of baseline daytime dozing was stronger for LHC than the other regions (Supplementary Table 6). Moreover, baseline insomnia symptom was associated with higher GM (1.04 ml;0.2%, 95% CI = 0.30 to 1.20, β estimate: 0.008) and WM (1.41 ml; 0.3%, 95% CI = 0.66 to 1.63, β estimate: 0.025) with the effect on WM being stronger than GM (Supplementary Table 6). No significant associations were detected between snoring and brain volumes.

### Sleep characteristics and cognition

Associations between sleep characteristics and cognitive functions are presented in Table 2 (refer to Supplementary Table 7 for full models). Although, no significant linear associations were detected between sleep duration and cognitive functions, significant quadratic associations were detected between sleep duration, VM and FI suggesting that both short and long sleep durations are associated with poorer performance on VM and FI. In addition, in categorical analyses, being a long sleeper (> 9 h/day) was associated with worse performance in VM (11.8%, 95% CI = 0.14 to 0.05, β estimate: 0.221), RT (3.4%, 95% CI = 5.72 to 34.40, β estimate: 0.20), NM (5.6%, 95% CI = −0.38 to −0.19, β estimate: −0.309) and FI (6.6%, 95% CI = −0.73 to −0.16, β estimate: −0.265) compared to being a normal sleeper. However, only being a short sleeper (< 6 h/day) was associated with lower FI performance (6.6%, 95% CI = −0.59 to −0.29, β estimate: −0.264). Based on the standardized β estimates, the effect of categorical sleep duration is stronger on VM (Supplementary Table 8).

**Table 2:** Association between cognitive functions and self-reported sleep duration (n = 14,206).

Independent variables		Dependent variables			
		Visual memory (number of incorrect matches)	Reaction time (correct answer time in ms)	Numeric memory (number of correct answers)	Fluid intelligence (pooled score)
Centred sleep duration^†^ (hours/day, 95% CI)		0.02 (−0.01, 0.05)	0.36 (−1.22, 1.94)	−0.02 (−0.04, −0.005)	−0.003 (−0.03, 0.003)
R^2^ adjusted		0.030	0.116	0.052	0.118
Categorical sleep duration ‡	Short	0.14 (−0.01, 0.30)	2.92 (−4.75, 10.56)	0.05 (−0.04, 0.15)	−0.44* (−0.59, −0.29)
	Long	0.42* (0.14, 0.05)	20.06* (5.72, 34.40)	−0.38* (−0.56, −0.19)	−0.44* (−0.73, −0.16)
R^2^ adjusted		0.031	0.119	0.049	0.120
Quadratic sleep duration		0.05* (0.03, 0.07)	0.89 (−0.09, 1.87)	−0.01 (−0.03, −0.002)	−0.07* (−0.09, −0.05)
R^2^ adjusted		0.031	0.117	0.052	0.121
Daytime dozing (yes/no, 95% CI)		0.07 (−0.01, 0.15)	8.05* (4.19, 11.90)	−0.04 (−0.09, 0.01)	−0.10* (−0.17, −0.02)
R^2^ adjusted		0.030	0.118	0.051	0.118
Snoring (yes/no, 95% CI)		0.08 (−0.002, 0.16)	1.02 (−2.38, 4.42)	−0.02 (−0.07, 0.002)	−0.01 (−0.08, 0.05)
R^2^ adjusted		0.010	0.117	0.051	0.118
Insomnia symptom (yes/no, 95% CI)		−0.004 (−0.08, 0.07)	−0.36 (−4.24, 3.53)	−0.04 (−0.01, 0.09)	0.03 (−0.04, 0.11)
R^2^ adjusted		0.030	0.116	0.051	0.118

The estimates are controlled for the following covariates: age, sex, education, body mass index, smoking, alcohol, physical activity, hypertension, and diabetes.

*95% CI* 95% confidence interval.

*p-value < 0.0125; p-value is considered significant after Bonferroni correction.

^†^Sleep duration is centred on 7 h of sleep per day.

^‡^Normal sleep duration refers to 6–9 h of sleep per day; normal sleep duration is the reference group; Short sleep duration refers to less than 6 h of sleep per day; long sleep duration refers to more than 9 h of sleep per day.

Further, experiencing daytime dozing was associated with worse performance in RT (1.4%, 95% CI = 4.19 to 11.90, β estimate: 0.076) and FI (1.5%, 95% CI = −0.17 to −0.02, β estimate: −0.033). The effect of daytime dozing on RT was stronger than on FI (Supplementary Table 8). No significant associations between snoring, insomnia symptom, and cognitive functions were found.

### Mediation effect of sleep on cognition through brain structure

Mediation analysis was considered for sleep duration, brain volumes and cognitive functions. However, the criteria were not met and therefore this analysis could not proceed (refer to Supplementary Table 9).

### Sensitivity analyses on subsample of participants with depression measures

Because depression is implicated in neurodegeneration^16^ and related to sleep problems^17^ sensitivity analyses on two subsamples of participants with (1) total data on sleep, brain volumes, and depression (n = 7528) and (2) complete data on sleep, cognitive functions, and depression (n = 3528) were conducted to investigate a possible contribution of depression symptomatology to the effects reported above (Supplementary Tables 10 and 11). Associations between sleep characteristics and brain volumes after controlling for depression remained essentially the same as those from the main analyses with small differences in statistical significance for sleep duration and hippocampus, which are likely attributable to the reduced statistical power. Similarly, the associations between sleep characteristics and cognitive functions showed similar findings although they did not reach significance for daytime dozing, NM, and FI.

### Sample size and power of detection

Due to the small effect sizes detected in the regression between sleep duration, LHC and RHC, the minimal detectable change was calculated for LHC and RHC using methods by Dupont et al. (1998)^18^. At 80% power, p = 0.05, and a sample size of 29,545 participants, the minimal detectable change for LHC was 0.007 ml and 0.006 ml per additional 1-h increase in sleep duration above 7 h/day at the baseline and the imaging visits, respectively. For RHC, at 80% power at 0.05 significance level with sample size of 29,545 participants, the minimal detectable change was 0.007 ml per additional 1-h increase in sleep duration above 7 h/day at both the baseline and imaging visits. These thresholds were lower than the effects detected in our analyses except an interaction between age and association between sleep duration and RHC at the imaging visit. This indicate that our results are mostly meaningful differences.

## Discussion

The main findings from this study were that poor sleep characteristics are associated with poorer brain health, and lower cognitive functions.

Sleep duration and dozing was consistently associated with impaired brain measures across most of the brain regions investigated. Indeed, longer sleep durations were associated with lower brain volumes with people sleeping 9 h/day having approximately 0.2% lower GM, 0.2% lower WM and 0.4–0.5% lower hippocampal volumes compared to people sleeping 7 h/day. On further investigation, this effect followed a U-shaped relationship with both shorter and longer sleep durations linked to lower GM and RHC. Thus, compared to people sleeping 6–9 h/day, those with both short (< 6 h/day) and long sleep durations (> 9 h/day) had 0.6% and 0.75% less GM respectively. These findings are consistent with previous studies that reported lower brain volumes in the elderly who sleep less than 7 hs^14^.

We also found daytime dozing to be associated with lower GM and LHC volume. Those who experienced daytime dozing had 0.2–0.3% lower GM and 0.3–0.4% lower LHC volume. This is in line with the literature reporting thinner cortex in daytime dozers^7,19^. Since in our sample daytime dozing was related to shorter sleep durations this effect may be attributable to the same underlying mechanisms as those experienced by short sleepers (see below).

Additionally, we found a paradoxical result indicating that the presence of insomnia symptom was associated with larger GM (0.2%) and WM (0.3%) volumes. This is consistent with previous findings suggesting that people suffering from insomnia have larger brain volumes in the anterior cingulate cortex and the left caudate nucleus^20,21^. However, other studies have reported contradictory results^21,22^. The reason for these differences is unclear. But it is possible that the global volumes investigated in the present study may have captured subtle differences spread across the whole brain, whereas more regional variability might have been highlighted through the investigation of regional structures (left orbital cortex, right middle temporal cortex, bilateral precuneus, posterior cingulate cortex and thalamus) in the other studies. It is also possible that methodological variation between these studies may explain the differences in findings. Indeed, while Altena et al. (2010)[22] and Winkelman et al. (2013)[20] used DSM-IV criteria, the Grau-Rivera et al. (2020)[21] investigated an epidemiological sample from the WHM survey. Moreover, there were important age differences, with inclusion of middle-aged adults in Altena et al. (2010) and Grau-Rivera et al. (2020) compared to young adults in Winkelman et al. (2013). Finally, since the presence of insomnia symptom was associated with shorter sleep duration it is possible that the decreased clearance of metabolic byproducts could be impaired and thus may have increased oxidative stress, and up-regulate pro-inflammatory pathways^5^. Upregulation of pro-inflammatory pathways may provoke a vasogenic oedema in the acute inflammatory stage which can cause a temporary increase in brain volumes in people with insomnia symptom^23^.

We also found that longer or shorter sleep duration and presence of daytime dozing were associated with lower cognition. Those who reported sleeping > 9 h/day had 11.8% lower visual memory, 3.4% slower reaction time, 5.6% lower numeric memory, and 6.6% lower fluid intelligence scores than those who slept 6–9 h/day. This is consistent with a previous meta-analysis indicating that both short and long sleep durations are associated with lower performance in multiple cognitive domains^15^. Similarly, daytime dozing was associated with slower reaction time (1.4%) and worse fluid intelligence (1.5%). This is also consistent with findings in the literature reporting greater risk of cognitive decline in people with daytime sleepiness^24^. However, unlike Kyle et al. (2017), we did not find any significant association between insomnia symptom and cognitive functions. This may be due to differences in sample size (n = 29,545 vs 477,529), or perhaps more likely given the substantial statistical power available in the present study, to socio-demographic or other participant differences between the cohorts.

Additionally, our findings are consistent with those of Tai et al. (2022)^25^ in that they also demonstrated quadratic sleep duration effects. However, the present study makes important new contributions by demonstrating how sleep characteristics such as daytime dozing, napping, insomnia symptom and snoring relate to brain volumes at midlife while also considering age and sex interactions. Moreover, it demonstrates that an optimal sleep duration window of 6–9 h previously identified in the literature is associated with better brain health. Importantly, analyses were controlled for multiple covariates and included sensitivity analyses, which revealed that the present results could not be better accounted for by an effect of depression. These findings are important as understanding how sleep characteristics interact and identifying optimal sleep duration at midlife can inform strategies to improve or maintain brain health in ageing.

The lower cognitive performances identified in short sleepers may be a consequence of lower brain volumes found in this group as multiple brain regions contribute to fluid intelligence^26^. However, we were unable to proceed with a planned mediation analysis to test this hypothesis since the necessary criteria were not met. This may indicate that brain volumes are not sufficiently sensitive measures to index subtle differences in performance on highly specific cognitive tasks attributable to sleep characteristics. An alternative explanation may be that increased accumulation of metabolites in the brain known to occur with insufficient sleep^8,27–30^, rather than brain atrophy, explains the observed lower cognitive functions associated with poorer sleep. This view is at least in part supported by other studies reporting a link between decreased brain clearance of amyloid β and tau, and cognitive decline^31,32^. In addition, short sleep durations are known to be associated with increased inflammation, which also contributes to cognitive decline^33^.

The mechanisms underlying the associations between sleep and brain health are not well understood. However, recent studies found short sleep to be linked to increased oxidative stress^34^, microglial activation^35^, elevated circulating and brain pro-inflammatory cytokines ^36^ implicated in neuronal injury^37^, decreased dendritic spine and density^38^, demyelination^39^, microgliosis^40^, and white matter lesions^41^ which contribute to brain atrophy^42,43^. Additionally, as noted above, the clearance of unwanted byproducts of cell metabolism, including amyloid β^5^ and tau, which is facilitated by the opening of the brain glymphatic channels during sleep, is likely to be insufficient below certain sleep duration and may lead to the accumulation of toxic metabolites^44^. This has been clearly demonstrated in sleep deprivation studies in which even isolated overnight sleep deprivation leads to elevated amyloid β^8,27^ and tau levels^28^. Cerebral accumulation of amyloid β is in turn associated with higher oxidative stress and neurodegeneration thus leading to brain atrophy^45,46^. Another plausible explanation is that short sleep increases the risk of depression^17^ which is known to lead to increased neuro-inflammation^47^ and to lower brain volumes^16^.

In contrast, the mechanisms through which long sleep duration is associated with lower brain volumes are less clear. The most probable explanation is reverse causality whereby the pathology underlying neurodegenerative or psychiatric conditions, which are known to be associated with lower brain volumes, are the cause and not the consequence of longer sleep duration. Indeed, as timing and duration of sleep are regulated by physiological responses to DNA damage, which builds up during wakeful periods^48^, DNA repair mechanisms impaired by pathological processes may signal to the brain sleep regulation centres that longer sleep duration is necessary. Similarly, depression is frequently associated with sleep disruptions and either shorter or longer sleep durations ^17^. Since depression is also known to be associated with lower brain volumes, it may explain the relationship between long sleep durations and lower volumes. However, the results of the sensitivity analyses conducted on subsamples of participants does not support such an effect in this study. Finally, sleep apnoea, which is associated with an increased risk of dementia^49^, cardiovascular diseases^50^, and diabetes^51^, may also partly explain the smaller brain volumes found in those sleeping more than 9 h/day.

The key strengths of the present study are its large neuroimaging cohort, inclusion of middle-aged healthy adults and adjustment of multiple confounding factors. A main limitation is the cross-sectional design of the study with neuroimaging data only available at one time-point. Other limitations include the use of self-reported measures of sleep characteristics which could be less accurate and subject to recall bias; the inclusion of relatively healthy participants which may have led to an underestimation of effects given lower rates of diabetes, smoking, hypertension which are known to be related to sleep characteristics and brain volumes^52^; the use of insomnia symptom characterised by “difficulty in falling asleep or waking up at night” which could be insufficient to clarify the link between insomnia and brain health.

In conclusion, this study provides important evidence indicating that poor sleep characteristics are linked to impaired brain health. Overall, these findings support the need for a greater focus on the contribution of sleep to brain health. They also highlight the need for further research into the development of interventions to improve sleep and the investigation of risk factors associated with poor sleep.

## Methods

### Participants

The UK Biobank study is a large population-based cohort study, which consists of 502,536 participants aged 37–73 years who were identified and invited through the National Health Service central registries. The methodology used has been extensively described elsewhere^53^. Briefly, those who accepted the invitation to participate undertook first assessment between 2006 and 2010 and were invited for first follow-up assessment between 2012 and 2014 and for imaging assessment in 2014 onwards. A large number of measures were collected at each assessment visit including demographic, dietary, health questionnaires, physical measures, blood samples and biomarkers and sub-sample of participants were also invited to undertake brain scans, cardiac scans and other imaging measures.

**Figure 1:**
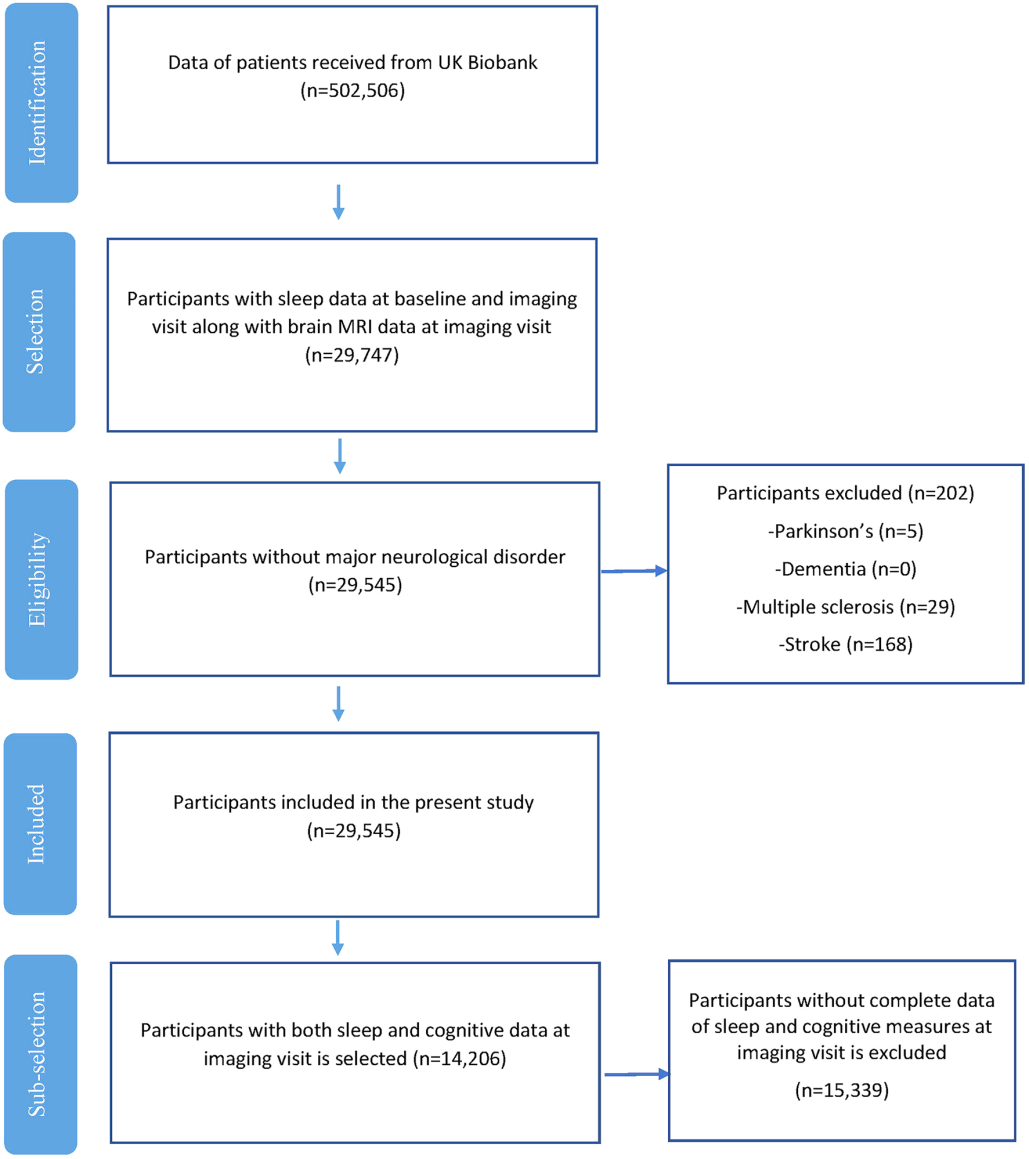
Flow chart of the participants’ selection process.

Participants who had complete data for sleep measures at both the baseline and the imaging visit and who undertook a structural magnetic resonance imaging (MRI) at the imaging visit were considered for inclusion (n = 29,747). Participants with major neurological disorders were excluded (stroke n = 168; dementia n = 0, Parkinson’s disease n = 5, and multiple sclerosis n = 29), leaving 29,545 participants for analyses focused on sleep and brain volumes (Fig. 1). Of those, 14,206 participants also had cognitive measures at the imaging visit and were included in the sleep-cognition analyses (Fig. 1). Only minor differences were detected between included and excluded participants (Supplementary Table 12).

Ethical approval was obtained from the North-West Multi-Centre Research Ethics Committee (11/NW/0382). All participants gave written informed consent. All methods were performed in accordance with the relevant guidelines and regulations from the UK Biobank and North-West Multi-Research Ethics committee.

### Sleep measures

Self-reported sleep characteristics were measured at the baseline, the first follow-up, and the imaging visits. They included sleep duration (hours), chronotype (morning person/more morning person/more evening person/evening person), difficulty getting up in the morning (not easy/fairly easy/easy/very easy), nap (rarely/sometimes/usually), daytime dozing (yes/no), snoring (yes/no), and insomnia symptom (yes/no) (Refer to Supplementary methods for sleep questionnaires). Choronotype was used as a measure of tendency a participant had to be more or less active or energetic at a certain time of the day. Sleep duration, daytime dozing, snoring, and insomnia symptom were chosen a priori as sleep characteristics of interest based on previous literature suggesting their possible association with brain health^7,14,15,21^. Sleep duration was investigated as a continuous variable as well as categorized into short sleepers (< 6 h/day), normal sleepers (6–9 h/day), and long sleepers (≥ 9 h/day)^54^. Presence of daytime dozing, and short/long sleep durations was considered poor sleep^14,15,19^.

### Brain scans

Brain scans were collected across three imaging centres on the same 3 T Siemens Skyra scanners using a 32-channel head coil for 5 min at imaging visit. Brain scan acquisition followed the protocol developed by UK Biobank^55^. The brain scans were processed and analysed by the UK Biobank team. In brief, T1-weighted brain MRI scans were acquired in sagittal orientation using a 3D magnetization-prepared rapid acquisition gradient echo sequence (resolution = 1 × 1 × 1 mm; matrix size = 208 × 256 × 256; T1/TR = 880/2000 ms)^55^.

### Segmentation and image analysis

Brain images were pre-processed by the UK Biobank using established protocols^55,56^. Acquired images were converted from DICOM to NIFTI followed by quality check, gradient distortion correction, cutting down field of view and defacing procedure. These images then underwent non-linear registration to MNI152 space using FNIRT (FMRIB's Nonlinear Image Registration Tool). The transformed brain images were tissue segmented using the Free surfer package (version 3.6.2)^57,58^. Region of interest (ROI) was selected based on the available literature suggesting possible vulnerability to sleep dysfunctions^7,14,59,60^. They included total grey matter volume (GM), total white matter volume (WM) and left and right hippocampus volumetric (LHC, RHC).

### Cognitive measures

Visual memory (VM), reaction time (RT), numeric memory (NM) and fluid intelligence (FI) were assessed through a touchscreen interface^61^. Visual memory was measured using a ‘pairs matching’ task. Reaction time was measured using a computerized ‘Snap’ game. Numeric memory was assessed through memorization of a x-digit number. Fluid intelligence was assessed through 13 questions designed to assess logic and reasoning ability independent of person’s acquired knowledge (see Supplementary method for details). The cognitive tests used in the UK Biobank study were found to be significantly correlated with previously validated standard tests, and to have moderate to high test–retest reliability^62^. The visual memory task was moderately associated with D-KEFS Tower Test (*r* = −0.40, *p* < 0.001). The reaction time task moderately correlated with DLRT Simple (*r* = 0.52, *p* < 0.001) The numeric memory task correlated significantly with several reference tests including WAIS-IV Digit Span Forwards, WAIS-IV Digit Span Backwards and WAIS-IV Digit Span Sequence [r = 0.43, 0.51, and 0.42, respectively (for all, *p* < 0.001)]. The fluid intelligence score correlated moderately with WAIS-IV Digits Span Forward (*r* = 0.37, *p* < 0.001), Backwards (*r* = 0.43, *p* < 0.001) and Sequence (*r* = 0.46, *p* < 0.001), and moderately with COGNITO Matrices (*r* = 0.38, *p* < 0.001) SDMT (*r* = 0.37, *p* < 0.001), PPVT (*r* = 0.36, *p* < 0.001), NIH Toolbox Picture vocabulary (*r* = 0.35, *p* < 0.001), WMS-IV Designs Total (*r* = 0.30, *p* < 0.001) and TMT part B (*r* = −0.30, *p* < 0.001)^62^.

### Covariates

Age, sex, education, smoking, alcohol intake (never, special occasions only, 1–3 times a month, once week, 3 times a week), body mass index (BMI, kg/m^2^), and diabetes were assessed by self-report. Physical activity levels (MET mins/week) were assessed with the International Physical Activity Questionnaire (IPAQ)^63^. Participants were classified as hypertensive if their blood pressure was ≥ 140 mmHg systolic or ≥ 90 mmHg diastolic, or if they reported taking blood pressure lowering medication. History of major depressive episodes was assessed by self-reported questionnaire based on the Patient health questionnaire that has been validated and performs adequately against PRIME-MD, a clinician administered criteria ^64,65^. Depression history, an important factor when considering associations between sleep, brain or cognition was not controlled in the main models because depression history measures were available for only 12–25.5% of the total participants. However, sensitivity analysis controlling for depression history on subsamples (n = 7528 for brain measures and n = 3528 for cognitive measures) were conducted.

### Statistical analyses

All statistical analyses were conducted using R version 3.6.2 under RStudio 1.2.5. Variables were checked for normality. Missing values (less than 1.9% of all variables) were imputed using Multivariate Imputation by Chained Equations (MICE) method^66^. Outliers with extreme values outside three-standard deviations were Windsorised using the following formula: mean + 3 × standard deviations + ((value − mean)/mean) to avoid undue influence while maintaining the relative order of data points^67–69^. Identified outlier were 63% of physical activity, 59.3% of visual memory performance, 1.1% of fluid intelligence score, 0.47% of numeric memory performance, 0.1–0.16% of sleep duration measures.

Several variables were centred to facilitate interpretation (age on 55 years; sleep duration on 7 hs). Hierarchical linear regression models were computed to assess the association between sleep characteristics, brain volumes, and cognition while controlling for age, sex, and intracranial volume (Model 1), and additionally, education, smoking, alcohol, physical activity, hypertension, and diabetes (Model 2). The α level was set at < 0.05. Bonferroni correction was applied to control for multiple comparisons and the adjusted p-value was 0.0125 (i.e., p = 0.05/4 comparisons).

Age and sex interactions along with non-linear associations (quadratic terms) were also tested (Model 3)^14,15^. Possible mediation of the sleep effects on cognition by brain volumes was investigated in 14,206 participants with complete sleep and cognition data with Baron and Kenny’s method (4 steps). The bootstrapping of indirect effect will be set to n = 1000. The effect of sleep on cognition was assessed in Step 1. The effect of sleep on brain volumes meeting criteria was assessed in Step 2. The effect of sleep on cognition with brain volumes and other covariates was assessed in Step 3. A causal mediation analysis was used to analyse the indirect effect of sleep on cognition through brain volumes in Step 4.

## Supplementary Information

Supplementary Information.

## Data Availability

The data that support the findings of this study are available from the UK Biobank, however restrictions apply to the availability of these data, which were used under license for the current study, and so are not publicly available. Data are however available from the authors upon reasonable request and with permission of the UK Biobank.
