# Supplementary material for: Poorer sleep impairs brain health at midlife
Source: Sci Rep. 2023 Feb 1;13:1874. doi: 10.1038/s41598-023-27913-9 (PMC9892039; doi:10.1038/s41598-023-27913-9)
Supplement: Supplementary file 1 — Supplementary Information. [file 41598_2023_27913_MOESM1_ESM.docx]

**Title** **Page**

**Title:** Poorer sleep impairs brain health at midlife

**Running Title:** Sleep and brain health

**Authors:**

Tergel Namsrai MD, MSc^1,^

Ananthan Ambikairajah PhD^1,2^

Nicolas Cherbuin PhD^1 *^

**Affiliations:**

1. Centre for Research on Ageing, Health and Wellbeing, Australian National University, Canberra, Australia^1^
2. Discipline of Psychology, Faculty of Health, University of Canberra, Canberra, ACT 2617, Australia^2^

**ORCID IDs:**

Tergel Namsrai: 0000-0002-7953-5299

Nicolas Cherbuin: 0000-0001-6481-074

Ananthan Ambikairajah: 0000-0002-5808-8020

***Correspondence:**

Nicolas Cherbuin, Centre for Research on Ageing, Health and Wellbeing, 54 Mills Road, Australian National University, Canberra, ACT 2601, Australia.

Tel: (612) 61253858; Fax: (612) 6125 3858; Email: nicolas.cherbuin@anu.edu.au

**Key words:** 1. Sleep, 2. Poor sleep 3. Brain volume, 4. Cognition, 5. Brain health, 6. UK Biobank

1. **Supplementary Tables**

This document contains supplementary tables containing information on study population characteristics, sleep and other covariates’ interaction, association between sleep duration and other sleep characteristics and mediation analysis.

**Supplementary table 1.**

| Supplementary table 1. Demographics and health characteristics of participants (n=29,545) | | | | |
| --- | --- | --- | --- | --- |
|  | **Total** | **Female** | **Male** | **p-value** |
| n | 29,545 | 15,780 | 13,765 |  |
| Demographics |  |  |  |  |
| Age at baseline visit (years)  Mean (SD) | 54.65 (7.44) | 53.98 (7.28) | 55.42 (7.55) | <0.001 |
| Age at imaging visit (years)  Mean (SD) | 63.0 (7.48) | 62.3 (7.32) | 63.8 (7.59) | <0.001 |
| Time until imaging visit (years)  Mean (SD) | 8.34 (1.63) | 8.35 (1.63) | 8.34 (1.64) | 0.73 |
| Education (%)  Higher | 6205 (21.0) | 3830 (24.3) | 2375 (17.3) | <0.001 |
| BMI at baseline visit (kg/m^2^)  mean (SD) | 26.50 (4.08) | 25.97 (4.37) | 27.11 (3.62) | <0.001 |
| BMI at imaging visit (kg/m^2^)  mean (SD) | 26.43 (4.25) | 25.97 (4.54) | 26.96 (3.82) | <0.001 |
| Smoking (%)  Never | 9793 (33.1) | 4846 (30.7) | 4947 (35.9) | <0.001 |
| Alcohol (%)  Never | 6661 (22.5) | 2883 (18.3) | 3778 (27.4) | <0.001 |
| Total MET minutes of physical activity (mins/week)  mean (SD) | 3.22 (0.44) | 3.22 (0.43) | 3.23 (0.45) | 0.029 |
| Hypertension  Yes (%) | 973 (3.3) | 972 (6.2) | 1 (0.0) | <0.001 |
| Diabetes  Yes (%) | 738 (2.5) | 267 (1.7) | 471 (3.4) | <0.001 |
| Sleep measures |  |  |  |  |
| Sleep duration at baseline visit (hours/day)  mean (SD) | 7.20 (0.93) | 7.24 (0.95) | 7.15 (0.91) | <0.001 |
| Sleep duration at imaging visit (hours/day)  mean (SD) | 7.16 (1.02) | 7.12 (1.03) | 7.20 (1.00) | <0.001 |
| Snoring at baseline visit (%)  Yes | 10760 (36.4) | 4194 (26.6) | 6566 (47.7) | <0.001 |
| Snoring at imaging visit (%)  Yes | 10821 (36.6) | 4581 (29.0) | 6240 (45.3) | <0.001 |
| Insomnia symptoms at baseline visit (%)  Yes | 21331 (72.2) | 12226 (77.5) | 9105 (66.1) | <0.001 |
| Insomnia symptoms at imaging visit (%)  Yes | 22950 (77.7) | 13187 (83.6) | 9763 (70.9) | <0.001 |
| Daytime dozing at baseline visit (%)  Yes | 5977 (20.2) | 2912 (18.5) | 3065 (22.3) | <0.001 |
| Daytime dozing at imaging visit (%)  Yes | 6781 (23.0) | 3178 (20.1) | 3603 (26.2) | NaN |
| Brain measures |  | | | |
| Grey matter volume (ml)  mean (SD) | 665.07 (59.35) | 634.65 (46.68) | 699.94 (52.78) | <0.001 |
| White matter volume (ml)  mean (SD) | 478.12 (57.20) | 450.91 (46.03) | 509.30 (52.65) | <0.001 |
| Intracranial volume (ml)  mean (SD) | 1547.62 (151.21) | 1466.30 (116.64) | 1640.84 (131.19) | <0.001 |
| Left hippocampus volume (ml)  mean (SD) | 3.96 (0.41) | 3.84 (0.36) | 4.10 (0.41) | <0.001 |
| Right hippocampus volume (ml)  mean (SD) | 4.13 (0.43) | 4.01 (0.38) | 4.27 (0.44) | <0.001 |
| Cognitive measures |  |  |  |  |
| Visual memory round 1 at imaging visit  mean (SD) | 0.62 (2.37) | 0.65 (2.37) | 0.59 (2.38) | 0.053 |
| Visual memory round 2 at imaging visit  mean (SD) | 3.56 (2.83) | 3.50 (2.72) | 3.63 (2.94) | 0.001 |
| Reaction time at imaging visit  mean (SD) | 589.37 (101.92) | 596.67 (101.21) | 581.13 (102.10) | <0.001 |
| Numeric memory at imaging visit  mean (SD) | 6.85 (1.27) | 6.76 (1.25) | 6.94 (1.29) | <0.001 |
| Fluid intelligence score at imaging visit  mean (SD) | 6.68 (2.05) | 6.55 (2.01) | 6.82 (2.10) | <0.001 |
|  | | | | |
|  | | | | |

**Supplementary table 2.**

| Supplementary table 2. Associations between sleep duration and demographics | | |
| --- | --- | --- |
|  | Dependent variable: | |
|  | **Sleep duration at baseline (hours/day)** | **Sleep duration at imaging (hours/day)** |
| Age at baseline visit  (Years, Estimate, SE) | -0.004^***^  (0.001) |  |
| Age at imaging visit  (Years, Estimate, SE) |  | 0.003^***^  (0.001) |
| Sex (male)  (Estimate, SE) | -0.087^***^  (0.011) | -0.075^***^  (0.017) |
| Age at baseline visit: Sex  (Estimate, SE) | 0.021^***^  (0.001) |  |
| Age at imaging visit: Sex  (Estimate, SE) |  | 0.018^***^  (0.002) |
| Adjusted R^2^ | 0.011 | 0.013 |
| BMI- Underweight at baseline visit  (kg/m^2^, Estimate, SE) | -0.267  (0.139) |  |
| BMI- Overweight at baseline visit  (kg/m^2^, Estimate, SE) | -0.031^**^  (0.012) |  |
| BMI- Obese at baseline visit  (kg/m^2^, Estimate, SE) | -0.091^***^  (0.016) |  |
| BMI- Underweight at baseline visit: Sex  (kg/m^2^, Estimate, SE) | 0.072  (0.439) |  |
| BMI- Overweight at baseline visit: Sex  (kg/m^2^, Estimate, SE) | -0.020  (0.025) |  |
| BMI- Obese at baseline visit: Sex  (kg/m^2^, Estimate, SE) | -0.034  (0.032) |  |
| BMI- Underweight at imaging visit  (kg/m^2^, Estimate, SE) |  | 0.064  (0.097) |
| BMI- Overweight at imaging visit  (kg/m^2^, Estimate, SE) |  | 0.019  (0.018) |
| BMI- Obese at imaging visit  (kg/m^2^, Estimate, SE) |  | 0.011^**^  (0.001) |
| BMI- Underweight at imaging visit: Sex  (Estimate, SE) |  | -0.047  (0.297) |
| BMI- Overweight at imaging visit: Sex  (Estimate, SE) |  | -0.058^**^  (0.027) |
| BMI- Obese at imaging visit: Sex  (Estimate, SE) |  | -0.093^***^  (0.034) |
| Adjusted R^2^ | 0.005 | 0.009 |
| SE, standard error  BMI, Body mass index  ^**^p<0.05; ^***^p<0.01 | | |

**Supplementary table 3.**

| Supplementary table 3. Associations between sleep duration, nap, getting up in the morning, daytime dozing, snoring, insomnia symptoms and chronotype | | |  |
| --- | --- | --- | --- |
|  | Dependent variable: | |  |
|  | Sleep duration at baseline | Sleep duration at imaging |  |
| Nap (Estimate, SE) | | |  |
| Nap sometimes at baseline visit | 0.167^**^  (0.012) |  |  |
| Nap usually at baseline visit | 0.592^***^  (0.030) |  |  |
| Nap sometimes at imaging visit |  | 0.105^***^  (0.019) |  |
| Nap usually at imaging visit |  | 0.586^***^  (0.047) |  |
| R2 adjusted | 0.023 | 0.021 |  |
| Difficulty getting up in the morning (Estimate, SE) | | |  |
| Getting up- Fairly easy at baseline visit | 0.058^***^  (0.018) |  |  |
| Getting up - Not very easy at baseline visit | 0.066^***^  (0.023) |  |  |
| Getting up - Not at all easy at baseline visit | 0.109^***^  (0.037) |  |  |
| Getting up- Fairly easy at imaging visit |  | 0.121^***^  (0.018) |  |
| Getting up- Not very easy at imaging visit |  | 0.128^***^  (0.027) |  |
| Getting up- Not at all easy at imaging visit |  | 0.141^***^  (0.045) |  |
| R2 adjusted | 0.006 | 0.011 |  |
| Daytime dozing (Yes/No; Estimate, SE) | | |  |
| Daytime dozing at baseline visit | -0.119^***^  (0.014) |  |  |
| Daytime dozing at imaging visit |  | -0.185^***^  (0.022) |  |
| Adjusted R2 | 0.008 | 0.012 |  |
| Snoring (Yes/No; Estimate, SE) | | |  |
| Snoring at baseline visit | 0.057^***^  (0.021) |  |  |
| Snoring at imaging visit |  | 0.082^***^  (0.021) |  |
| Adjusted R2 | 0.006 | 0.010 |  |
| Insomnia symptoms (Yes/No; Estimate, SE) | | |  |
| Insomnia symptoms at baseline visit | -0.232^***^  (0.027) |  |  |
| Insomnia symptoms at imaging visit |  | -0.363^***^  (0.031) |  |
| Adjusted R2 | 0.022 | 0.031 |  |
| Chronotype (Estimate, SE) | | |  |
| Chronotype- Evening at baseline visit | 0.111^***^  (0.020) |  |  |
| Chronotype- More morning at baseline visit | 0.083^***^  (0.020) |  |  |
| Chronotype- More evening at baseline visit | 0.015  (0.021) |  |  |
| Chronotype- Evening at imaging visit |  | 0.083^***^  (0.022) |  |
| Chronotype- More morning at imaging visit |  | 0.031  (0.021) |  |
| Chronotype- More evening at imaging visit |  | -0.102^***^  (0.022) |  |
| Adjusted R2 | 0.007 | 0.014 |  |
| SE, standard error  ^**^p<0.05; ^***^p<0.01 | | | |

**Supplementary table 4.**

| Supplementary table 4. Association between sleep duration and brain volumes | | | |  |
| --- | --- | --- | --- | --- |
|  | | | |  |
|  | *Dependent variable:* | | |  |
|  |  | | |  |
|  | **GM** | | |  |
|  | Model 1 | Model2 | Model 1 | Model 2 |
|  |  |  |  |  |
| Sleep duration at baseline visit  (Hours/day, Estimate, SE) | -0.73^***^ | -0.77^***^ |  |  |
|  | (0.18) | (0.18) |  |  |
| Sleep duration at imaging visit  (Hours/day, Estimate, SE) |  |  | -0.27 | -0.31 |
|  |  |  | (0.17) | (0.17) |
| Observations | 29,545 | 29,545 | 29,545 | 29,545 |
| R^2^ | 0.76 | 0.76 | 0.76 | 0.76 |
| Adjusted R^2^ | 0.76 | 0.76 | 0.76 | 0.76 |
|  | | | |  |
|  | *Dependent variable:* | | |  |
|  |  | | |  |
|  | **WM** | | |  |
|  | Model 1 | Model 2 | Model 1 | Model 2 |
|  |  |  |  |  |
| Sleep duration at baseline visit  (Hours/day, Estimate, SE) | -0.52^***^ |  | -0.57^***^ |  |
|  | (0.18) |  | (0.18) |  |
| Sleep duration at imaging visit  (Hours/day, Estimate, SE) |  | -0.48^***^ |  | -0.51^***^ |
|  |  | (0.17) |  | (0.17) |
|  |  |  | (0.0001) | (0.0001) |
|  |  |  |  |  |
| Observations | 29,545 | 29,545 | 29,545 | 29,545 |
| R^2^ | 0.74 | 0.74 | 0.74 | 0.74 |
| Adjusted R^2^ | 0.74 | 0.74 | 0.74 | 0.74 |
|  | | | |  |
|  | *Dependent variable:* | | |  |
|  |  | | |  |
|  | LHC | | |  |
|  | Model 1 | Model 2 | Model 1 | Model 2 |
|  |  |  |  |  |
| Sleep duration at baseline visit  (Hours/day, Estimate, SE) | -0.01^***^ |  | -0.01^***^ |  |
|  | (0.002) |  | (0.002) |  |
| Sleep duration at imaging visit  (Hours/day, Estimate, SE) |  | -0.01^***^  (0.002) |  | -0.01^***^  (0.002) |
|  |  |  |  |  |
| Observations | 29,545 | 29,545 | 29,545 | 29,545 |
| R^2^ | 0.36 | 0.37 | 0.37 | 0.37 |
| Adjusted R^2^ | 0.36 | 0.37 | 0.37 | 0.37 |
|  | *Dependent variable:* | | |  |
|  | **RHC** | | |  |
|  | Model 1 | Model 2 | Model 1 | Model 2 |
|  |  |  |  |  |
| Sleep duration at baseline visit  (Hours/day, Estimate, SE) | -0.01^***^ |  | -0.01^***^ |  |
|  | (0.002) |  | (0.002) |  |
| Sleep duration at imaging visit  (Hours/day, Estimate, SE) |  | -0.01^***^ |  | -0.01^***^ |
|  |  | (0.002) |  | (0.002) |
| Observations | 29,545 | 29,545 | 29,545 | 29,545 |
| R^2^ | 0.35 | 0.36 | 0.35 | 0.36 |
| Adjusted R^2^ | 0.35 | 0.36 | 0.35 | 0.36 |
|  | *Dependent variable:* | | |  |
|  | **GM** | | |  |
|  | Model 1 | Model 2 | Model 1 | Model 2 |
|  |  |  |  |  |
| Categorical sleep duration at baseline visit – Short sleepers  (Hours/day, Estimate, SE) | -2.51^**^ (1.01) |  | -1.76 (1.01) |  |
| Categorical sleep duration at baseline visit – Long sleepers  (Hours/day, Estimate, SE) | -5.67^***^ (1.44) |  | -4.13^***^ (1.43) |  |
| Categorical sleep duration at imaging visit – Short sleepers  Estimate, SE) |  | -4.58^***^ (0.81) |  | -3.74^***^  (0.81) |
| Categorical sleep duration at imaging visit – Long sleepers  (Hours/day, Estimate, SE) |  | -6.45^***^ (1.45) |  | -4.98^***^  (1.45) |
| Observations | 22,791 | 22,401 | 22,791 | 22,401 |
| R^2^ | 0.76 | 0.76 | 0.76 | 0.77 |
| Adjusted R^2^ | 0.76 | 0.76 | 0.76 | 0.77 |
|  | *Dependent variable:* | | |  |
|  | **WM** | | |  |
|  | Model 1 | Model 2 | Model 1 | Model 2 |
|  |  |  |  |  |
| Categorical sleep duration at baseline visit – Short sleepers  (Hours/day, Estimate, SE) | 0.41 (1.01) |  | 1.29 (1.01) |  |
| Categorical sleep duration at baseline visit – Long sleepers  (Hours/day, Estimate, SE) | -2.84^**^ (1.44) |  | -1.22 (1.44) |  |
| Categorical sleep duration at imaging visit – Short sleepers  Estimate, SE) |  | -0.81 (0.82) |  | 0.22 (0.82) |
| Categorical sleep duration at imaging visit – Long sleepers  (Hours/day, Estimate, SE) |  | -4.18^***^ (1.46) |  | -2.53 (1.45) |
| Observations | 22,791 | 22,401 | 22,791 | 22,401 |
| R^2^ | 0.74 | 0.74 | 0.74 | 0.74 |
| Adjusted R^2^ | 0.74 | 0.74 | 0.74 | 0.74 |
|  | *Dependent variable:* | | |  |
|  | **LHC** | | |  |
|  | Model 1 | Model 2 | Model 1 | Model 2 |
|  |  |  |  |  |
| Categorical sleep duration at baseline visit – Short sleepers  (Hours/day, Estimate, SE) | 0.01 (0.01) |  | 0.01 (0.01) |  |
| Categorical sleep duration at baseline visit – Long sleepers  (Hours/day, Estimate, SE) | -0.02 (0.02) |  | -0.02 (0.02) |  |
| Categorical sleep duration at imaging visit – Short sleepers  Estimate, SE) |  | -0.01 (0.01) |  | -0.01 (0.01) |
| Categorical sleep duration at imaging visit – Long sleepers  (Hours/day, Estimate, SE) |  | -0.05^***^  (0.02) |  | -0.05^***^  (0.02) |
| Observations | 22,791 | 22,401 | 22,791 | 22,401 |
| R^2^ | 0.36 | 0.37 | 0.37 | 0.37 |
| Adjusted R^2^ | 0.36 | 0.37 | 0.37 | 0.37 |
|  | *Dependent variable:* | | |  |
|  | **RHC** | | |  |
|  | Model 1 | Model 2 | Model 1 | Model 2 |
|  |  |  |  |  |
| Categorical sleep duration at baseline visit – Short sleepers  (Hours/day, Estimate, SE) | -0.01 |  | -0.01 |  |
| Categorical sleep duration at baseline visit – Long sleepers  (Hours/day, Estimate, SE) | (0.01) |  | (0.01) |  |
| Categorical sleep duration at imaging visit – Short sleepers  Estimate, SE) | -0.02 |  | -0.02 |  |
| Categorical sleep duration at imaging visit – Long sleepers  (Hours/day, Estimate, SE) | (0.02) |  | (0.02) |  |
| Categorical sleep duration at baseline visit – Short sleepers  (Hours/day, Estimate, SE) |  | -0.02 |  | -0.02 |
| Categorical sleep duration at baseline visit – Long sleepers  (Hours/day, Estimate, SE) |  | (0.01) |  | (0.01) |
| Categorical sleep duration at imaging visit – Short sleepers  Estimate, SE) |  | -0.06^***^ |  | -0.06^***^ |
|  |  | (0.02) |  | (0.02) |
| Observations | 22,791 | 22,401 | 22,791 | 22,401 |
| R^2^ | 0.35 | 0.36 | 0.35 | 0.36 |
| Adjusted R^2^ | 0.35 | 0.36 | 0.35 | 0.36 |
|  | *Dependent variable:* | | |  |
|  | **GM** | | |  |
|  | Model 1 | Model 2 | Model 1 | Model 2 |
|  |  |  |  |  |
| (Sleep duration at baseline)^2^ | -0.78^***^ |  | -0.57^***^ |  |
|  | (0.13) |  | (0.13) |  |
| (Sleep duration at follow-up)^2^ |  | -0.90^***^ |  | -0.73^***^ |
|  |  | (0.10) |  | (0.10) |
| Observations | 29,545 | 29,545 | 29,545 | 29,545 |
| R^2^ | 0.76 | 0.76 | 0.76 | 0.77 |
| Adjusted R^2^ | 0.76 | 0.76 | 0.76 | 0.76 |
|  | *Dependent variable:* | | |  |
|  |  | | |  |
|  | **WM** | | |  |
|  | Model 1 | Model 2 | Model 1 | Model 2 |
|  |  |  |  |  |
| (Sleep duration at baseline)^2^ | -0.25 (0.13) |  | -0.04 (0.13) |  |
| (Sleep duration at follow-up)^2^ |  | -0.28^***^ (0.10) |  | -0.09 (0.10) |
| Observations | 29,545 | 29,545 | 29,545 | 29,545 |
| R^2^ | 0.74 | 0.74 | 0.74 | 0.74 |
| Adjusted R^2^ | 0.74 | 0.74 | 0.74 | 0.74 |
|  | *Dependent variable:* | | |  |
|  | **LHC** | | |  |
|  | Model 1 | Model 2 | Model 1 | Model 2 |
| (Sleep duration at baseline)^2^ | -0.002  (0.001) |  | -0.002  (0.001) |  |
| (Sleep duration at follow-up)^2^ |  | -0.005^***^  (0.001) |  | -0.004^***^  (0.001) |
| Observations | 29,545 | 29,545 | 29,545 | 29,545 |
| R^2^ | 0.36 | 0.37 | 0.37 | 0.37 |
| Adjusted R^2^ | 0.36 | 0.37 | 0.37 | 0.37 |
|  | *Dependent variable:* | | | |
|  | **RHC** | | | |
|  | Model 1 | Model 2 | Model 1 | Model 2 |
|  |  |  |  |  |
| (Sleep duration at baseline)^2^ | -0.005^***^  (0.002) | -0.005^***^  (0.002) |  |  |
| (Sleep duration at follow-up)^2^ |  |  | -0.01^***^ (0.001) | -0.005^***^   (0.001) |
| Observations | 29,545 | 29,545 | 29,545 | 29,545 |
| R^2^ | 0.35 | 0.36 | 0.35 | 0.36 |
| Adjusted R^2^ | 0.35 | 0.36 | 0.35 | 0.36 |
|  |  |  |  |  |
| SE, standard error  P<0.05 not controlled for multiple comparison  ^**^p<0.05; ^***^p<0.01 | | | | |

**Supplementary table 5.**

| Supplementary table 5. Interaction between sleep duration, age, sex and brain volumes | | | | |
| --- | --- | --- | --- | --- |
|  | *Dependent variable:* | | | |
|  | **GM (ml)** | **WM (ml)** | **LHC (ml)** | **RHC (ml)** |
|  |  |  |  |  |
| Sleep duration at baseline visit  (hours/day) | -0.48^**^  (-0.95, -0.004) | -0.56^**^  (-1.03,  -0.08) | -0.01^**^  (-0.01,  -0.0003) | -0.01  (-0.01, 0.0003) |
| Sleep duration at baseline visit: age at baseline visit  (hours/day, years, 95% CI) | 0.02  (-0.03, 0.07) | -0.03  (-0.078, 0.018) | 0.0002  (-0.078, 0.018) | -0.000  (-0.001, 0.001) |
| Sleep duration at baseline visit: sex  (hours/day, male, 95% CI) | -0.68  (-1.40, 0.03) | -0.015  (-0.734, 0.704) | -0.01^**^  (-0.02, -0.001) | -0.01^**^  (-0.02, -0.001) |
| Sleep duration at imaging visit (hours/day) | 0.66^**^  (0.12, 1.20) | 0.19  (-0.36, 0.73) | 0.002  (-0.004, 0.01) | 0.003  (-0.004, 0.01) |
| Sleep duration at imaging visit: age at imaging visit  (hours/day, years, 95% CI) | -0.08 ^**^  (-0.13, -0.04) | -0.07 ^**^  (-0.113, -0.026) | 0.001  (-0.001, -0.0001) | -0.001^***^  (-0.001, -0.0004) |
| Sleep duration at imaging visit: sex  (hours/day, male, 95% CI) | -0.61  (-1.12, 0.05) | -0.26  (-0.917, 0.395) | -0.01^**^  (-0.002, -0.001) | -0.01  (-0.01, 0.002) |
| CI, confidence interval set at 95%  ^**^p<0.05; ^***^p<0.01 | | | | |

**Supplementary table 6.**

| **Supplementary table 6. Associations between sleep characteristics and standardized brain volumes (n=29,545).** | | | | | | | | | |
| --- | --- | --- | --- | --- | --- | --- | --- | --- | --- |
|  | | | | | Dependent variable | | | | |
| Independent variables | | | | | **Standardized GM**  **(ml)** | **Standardized WM**  **(ml)** | **Standardized LHC**  **(ml)** | | **Standardized RHC**  **(ml)** |
| Sleep duration  (hours/day, CI) | | Baseline | | | -0.012^*^  (-0.018,  -0.007) | -0.009  (-0.015,  -0.004) | -0.022^*^  (-0.031,  -0.013) | | -0.021^*^  (-0.030,  -0.012) |
|  |  | Imaging | | | -0.005  (-0.011,  -0.003) | -0.009^*^  (-0.015,  -0.003) | -0.02^*^  (-0.03,  -0.008) | | -0.017^*^  (-0.026,  -0.008) |
| Adjusted R^2^ | Baseline | | | | 0.765 | 0.742 | 0.365 | | 0.354 |
|  | Imaging | | | | 0.765 | 0.744 | 0.369 | | 0.357 |
| Categorical sleep duration | | Baseline | | Short | -0.030  (-0.063, 0.004) | 0.023  (-0.012,  0.057) | 0.029  (-0.026,  0.083) | | -0.019  (-0.074,  0.036) |
|  |  |  |  | Long | -0.070^*^  (-0.117,  -0.022) | -0.021  (-0.071,  0.028) | -0.040  (-0.117,  0.038) | | -0.056  (-0.134,  0.023) |
|  |  | Imaging | Short | | -0.063^*^  (-0.090,  -0.036) | 0.004  (-0.024,  0.032) | -0.025  (-0.069,  0.019) | | -0.036  (-0.080,  0.009) |
|  |  |  | Long | | -0.084^*^  (-0.132,  -0.036) | -0.044  (-0.094,  0.006) | -0.113^*^  (-0.019,  -0.034) | | -0.144^*^  (-0.224,  -0.065) |
| Adjusted R^2^ | | Baseline | | | 0.764 | 0.741 | 0.365 | | 0.354 |
|  |  | Imaging | | | 0.765 | 0.743 | 0.369 | | 0.359 |
| Daytime dozing  (Yes/No, CI) | | Baseline | | | -0.03^*^  (-0.04,  -0.02) | -0.017  (-0.03,  -0.002) | -0.02^*^  (-0.02,  -0.01) | | -0.029  (-0.052,  -0.006) |
|  |  | Imaging | | | -0.028^*^  (-0.041,  -0.014) | -0.021^*^  (-0.035,  -0.007) | -0.031^*^  (-0.053,  -0.010) | | -0.002  (-0.047,  -0.003) |
| Adjusted R^2^ | Baseline | | | | 0.765 | 0.320 | 0.365 | | 0.354 |
|  | Imaging | | | | 0.765 | 0.321 | 0.369 | | 0.357 |
| Snoring  (Yes/No, CI) | | Baseline | | | -0.003  (-0.015,  0.010) | 0.001  (-0.012,  0.013) | -0.001  (-0.021, 0.019) | | 0.004  (-0.016, 0.025) |
|  |  | Imaging | | | 0.009  (-0.003,  0.020) | 0.004  (-0.009,  0.016) | 0.004  (0.009,  0.016) | | 0.023  (0.003, 0.043) |
| Adjusted R^2^ | Baseline | | | | 0.764 | 0.742 | 0.365 | | 0.365 |
|  | Imaging | | | | 0.765 | 0.744 | 0.369 | | 0.369 |
| Insomnia symptoms  (Yes/No, CI) | | Baseline | | | 0.018^*^  (0.005,  0.030) | 0.025^*^  (0.012,  0.038) | 0.021  (0.0001,  0.041) | | 0.020  (-0.001,  0.041) |
|  |  | Imaging | | | 0.007  (-0.007,  0.20) | 0.015  (0.001,  0.029) | 0.024  (0.002,  0.046) | | 0.031^*^  (0.009,  0.054) |
| Adjusted R^2^ | Baseline | | | | 0.765 | 0.743 | 0.365 | | 0.353 |
|  | Imaging | | | | 0.765 | 0.744 | 0.369 | 0.357 | |
| 95% CI, 95% confidence interval  *p-value <0.0125; p-value is considered significant after Bonferroni correction  † Sleep duration is centred on 7 hours of sleep per day  ‡ Normal sleep duration refers to 6-9 hours of sleep per day; Normal sleep duration is the reference group; Short sleep duration refers to less than 6 hours of sleep per day; Long sleep duration refers to more than 9 hours of sleep per day  The estimates are controlled for the following covariates: age, sex, education, body mass index, smoking, alcohol, physical activity, hypertension, and diabetes | | | | | | | | | |

**Supplementary table 7.**

| Supplementary table 7. Association between sleep duration and cognitive functions at imaging visit | | |
| --- | --- | --- |
|  | | |
|  | *Dependent variable:* | |
|  |  | |
|  | **Visual memory at imaging visit** | |
|  | Model 1 | Model 2 |
|  |  |  |
| Sleep duration at imaging visit  (hours/day, Estimate, SE) | 0.02 | 0.02 |
|  | (0.02) | (0.02) |
| Observations | 14,206 | 14,206 |
| R^2^ | 0.03 | 0.03 |
| Adjusted R^2^ | 0.03 | 0.03 |
|  | *Dependent variable:* | |
|  |  | |
|  | **Reaction time at imaging visit** | |
|  | Model 1 | Model 2 |
|  |  |  |
| Sleep duration at imaging visit  (hours/day, Estimate, SE) | 0.26 (0.81) | 0.36 (0.81) |
| Observations | 14,206 | 14,206 |
| R^2^ | 0.11 | 0.12 |
| Adjusted R^2^ | 0.11 | 0.12 |
|  | *Dependent variable:* | |
|  |  | |
|  | **Numeric memory at imaging visit** | |
|  | Model 1 | Model 2 |
|  |  |  |
| Sleep duration at imaging visit  (hours/day, Estimate, SE) | -0.02^**^ | -0.02^**^ |
|  | (0.01) | (0.01) |
| Observations | 14,206 | 14,206 |
| R^2^ | 0.04 | 0.05 |
| Adjusted R^2^ | 0.04 | 0.05 |
|  | *Dependent variable:* | |
|  |  | |
|  | **Fluid intelligence at imaging visit** | |
|  | Model 1 | Model 2 |
|  |  |  |
| Sleep duration at imaging visit  (hours/day, Estimate, SE) | 0.01 | -0.003 |
|  | (0.02) | (0.02) |
| Observations | 14,206 | 14,206 |
| R^2^ | 0.10 | 0.12 |
| Adjusted R^2^ | 0.10 | 0.12 |
|  | *Dependent variable:* |  |
|  |  |  |
|  | **Visual memory at imaging visit** | |
|  | Model 1 | Model 2 |
|  |  |  |
| Categorical sleep duration at imaging visit- short sleepers  (hours/day, Estimate, SE) | 0.14 | 0.15 |
|  | (0.08) | (0.08) |
| Categorical sleep duration at imaging visit- long sleepers  (hours/day, Estimate, SE) | 0.41^***^ | 0.42^***^ |
| Observations | 10,869 | 10,869 |
| R^2^ | 0.03 | 0.03 |
| Adjusted R^2^ | 0.03 | 0.03 |
|  | *Dependent variable:* | |
|  |  | |
|  | **Reaction time at imaging visit** | |
|  | Model 1 | Model 2 |
|  |  |  |
| Categorical sleep duration at imaging visit- short sleepers  (hours/day, Estimate, SE) | 4.67 | 2.92 |
|  | (3.88) | (3.90) |
| Categorical sleep duration at imaging visit- long sleepers  (hours/day, Estimate, SE) | 21.80^***^ | 20.06^***^ |
|  | (7.31) | (7.32) |
| Observations | 10,869 | 10,869 |
| R^2^ | 0.12 | 0.12 |
| Adjusted R^2^ | 0.12 | 0.12 |
|  | *Dependent variable:* | |
|  |  | |
|  | **Numeric memory at imaging visit** | |
|  | Model 1 | Model 2 |
|  |  |  |
| Categorical sleep duration at imaging visit- short sleepers  (hours/day, Estimate, SE) | 0.02 | 0.05 |
|  | (0.05) | (0.05) |
| Categorical sleep duration at imaging visit- long sleepers  (hours/day, Estimate, SE) | -0.39^***^ | -0.38^***^ |
|  | (0.09) | (0.09) |
| Observations | 10,869 | 10,869 |
| R^2^ | 0.04 | 0.05 |
| Adjusted R^2^ | 0.04 | 0.05 |
|  | *Dependent variable:* | |
|  |  | |
|  | **Fluid intelligence at imaging visit** | |
|  | Model 1 | Model 2 |
|  |  |  |
| Categorical sleep duration at imaging visit- short sleepers  (hours/day, Estimate, SE) | -0.51^***^ | -0.44^***^ |
|  | (0.08) | (0.08) |
| Categorical sleep duration at imaging visit- long sleepers  (hours/day, Estimate, SE) | -0.46^***^ | -0.44^***^ |
|  | (0.15) | (0.15) |
| Observations | 10,869 | 10,869 |
| R^2^ | 0.11 | 0.12 |
| Adjusted R^2^ | 0.11 | 0.12 |
|  |  |  |
|  | *Dependent variable:* | |
|  |  | |
|  | **Visual memory at imaging visit** | |
|  | Model 1 | Model 2 |
|  |  |  |
| (Sleep duration at follow-up)^2^ | 0.05^***^ (0.01) | 0.05^***^ (0.01) |
| Observations | 14,206 | 14,206 |
| R^2^ | 0.03 | 0.03 |
| Adjusted R^2^ | 0.03 | 0.03 |
|  | *Dependent variable:* | |
|  |  | |
|  | **Reaction time at imaging visit** | |
|  | Model 1 | Model 2 |
|  |  |  |
| (Sleep duration at follow-up)^2^ | 1.14^**^ | 0.89 |
|  | (0.50) | (0.50) |
| Observations | 14,206 | 14,206 |
| R^2^ | 0.12 | 0.12 |
| Adjusted R^2^ | 0.11 | 0.12 |
|  | *Dependent variable:* | |
|  |  | |
|  | **Numeric memory at imaging visit** | |
|  | Model 1 | Model 2 |
|  |  |  |
| (Sleep duration at follow-up)^2^ | -0.02^***^ | -0.01^**^ |
|  | (0.01) | (0.01) |
| Observations | 14,206 | 14,206 |
| R^2^ | 0.04 | 0.05 |
| Adjusted R^2^ | 0.04 | 0.05 |
|  | *Dependent variable:* | |
|  |  | |
|  | **Fluid intelligence at imaging visit** | |
|  | Model 1 | Model 2 |
|  |  |  |
| (Sleep duration at follow-up)^2^ | -0.08^***^ | -0.07^***^ |
|  | (0.01) | (0.01) |
| Observations | 14,206 | 14,206 |
| R^2^ | 0.11 | 0.12 |
| Adjusted R^2^ | 0.11 | 0.12 |
| SE, standard error  P<0.05 not controlled for multiple comparison  ^**^p<0.05; ^***^p<0.01 | | |

**Supplementary table 8.**

| **Supplementary table 8. Association between sleep duration and standardized cognitive functions (n=14,206).** | | | | | |
| --- | --- | --- | --- | --- | --- |
|  | | Dependent variable | | | |
| Independent variables | | **Visual memory**  **(Number of incorrect matches)** | **Reaction time**  **(Correct answer time in ms)** | **Numeric memory**  **(Number of correct answers)** | **Fluid intelligence**  **(pooled score)** |
| Sleep duration†  (hours/day, CI) | | 0.010  (-0.006, 0.026) | 0.003  (-013, 0.018) | -0.018  (-0.034, -0.002) | 0.002  (-0.014, 0.018) |
| R^2^ adjusted | | 0.030 | 0.116 | 0.052 | 0.118 |
| Categorical sleep duration | Short | 0.078  (0.001, 0.156) | 0.038  (-0.036, 0.113) | 0.030  (-0.047, 0.107) | -0.264^*^  (-0.341, -0.187) |
|  | Long | 0.221^*^  (0.076, 0.366) | 0.206^*^  (0.066, 0.346) | -0.309^*^  (-0.454,  -0.163) | -0.265^*^  (-0.410,  -0.121) |
| R^2^ adjusted | | 0.031 | 0.119 | 0.049 | 0.120 |
| Daytime dozing  (Yes/No, CI) | | 0.035  (-0.005, 0.074) | 0.076^*^  (0.038, 0.113) | -0.026  (-0.065, 0.013) | -0.033^*^  (-0.072, -0.006) |
| R^2^ adjusted | | 0.030 | 0.118 | 0.051 | 0.118 |
| Snoring  (Yes/No, CI) | | 0.020  (-0.014, 0.055) | 0.013  (-0.020, 0.047) | -0.024  (-0.059, 0.010) | -0.026  (-0.060, 0.008) |
| R^2^ adjusted | | 0.010 | 0.117 | 0.051 | 0.118 |
| Insomnia symptoms  (Yes/No, CI) | | -0.001  (-0.041, 0.038) | -0.002  (-0.040, 0.036) | 0.030  (-0.010, 0.069) | 0.007  (-0.032, 0.046) |
| R^2^ adjusted | | 0.030 | 0.116 | 0.051 | 0.118 |
| 95% CI, 95% confidence interval  *p-value <0.0125; p-value is considered significant after Bonferroni correction  † Sleep duration is centred on 7 hours of sleep per day  ‡ Normal sleep duration refers to 6-9 hours of sleep per day; Normal sleep duration is the reference group; Short sleep duration refers to less than 6 hours of sleep per day; Long sleep duration refers to more than 9 hours of sleep per day  The estimates are controlled for the following covariates: age, sex, education, body mass index, smoking, alcohol, physical activity, hypertension, and diabetes | | | | | |

**Supplementary table 9.**

| Supplementary table 9. **Association of sleep duration on brain volumes at imaging visit** | | | | |
| --- | --- | --- | --- | --- |
|  | | |  |  |
|  | *Dependent variable:* | | | |
|  | **GM (ml)** | **WM (ml)** | **LHC (ml)** | **RHC (ml)** |
|  |  |  |  |  |
| Sleep duration at imaging visit  (hours/day, (95% CI) | -0.117  (-0.586, 0.35) | -0.394  (-0.867, 0.079) | -0.005  (-0.010, 0.0001) | -0.006  (-0.012, -0.001) |
|  |  |  |  |  |
| ^**^p<0.05; ^***^p<0.01 | | | | |

**Supplementary table 10.**

| **Supplementary table 10. Sensitivity analysis on the association between sleep characteristics and brain volumes on participants with available depression measure (n=7,528).** | | | | | | | | |
| --- | --- | --- | --- | --- | --- | --- | --- | --- |
|  | | | | Dependent variable | | | | |
| Independent variables | | | | **GM**  **(ml)** | **WM**  **(ml)** | **LHC**  **(ml)** | | **RHC**  **(ml)** |
| Centred sleep duration †  (hours/day, 95% CI) | | Baseline | | -1.04^*^  (-1.74,  -0.34) | -0.77  (-1.47,  -0.07) | -0.01  (-0.01,  0.01) | | -0.01  (-0.02,  0.001) |
|  |  | Imaging | | -0.44  (-1.09, 0.20) | -0.85^*^  (-1.49,-0.20) | -0.002  (-0.01,  0.01) | | -0.003  (-0.01,  0.005) |
| Adjusted R^2^ | Baseline | | | 0.76 | 0.74 | 0.35 | | 0.34 |
|  | Imaging | | | 0.76 | 0.74 | 0.35 | | 0.34 |
| Categorical sleep duration‡  (95% CI) | | Baseline | Short | -3.32  (-7.37, 0.73) | -2.53  (-1.55,  6.61) | -0.04  (-0.08,  0.01) | | -0.08^*^  (-0.13,  -0.03) |
|  |  |  | Long | -5.64^*^  (-11.07,  -0.22) | -4.41  (-9.88,  1.05) | -0.03  (-0.10,  0.03) | | -0.03  (-0.09,  0.04) |
|  |  | Imaging | Short | -4.98^*^  (-8.09, -1.88) | 0.03  (-3.07,  3.13) | 0.04  (-0.08,  -0.01) | | -0.06^*^  (-0.09,  -0.02) |
|  |  |  | Long | -5.41^*^  (-10.85, -10.02) | -2.61  (-8.05,  2.83) | -0.07  (-0.13,  -0.01) | | -0.09^*^  (-0.16,  -0.03) |
| Adjusted R^2^ | Baseline | | | 0.76 | 0.74 | 0.34 | | 0.35 |
|  | Imaging | | | 0.76 | 0.74 | 0.35 | | 0.36 |
| Quadratic sleep duration  (95% CI) | | Baseline | | -0.87^*^  (-1.37,  -0.38) | -0.14  (-0.63,  0.36) | -0.01  (-0.01,  -0.001) | | -0.01^*^  (-0.02,  -0.003) |
|  |  | Imaging | | -0.95^*^  (-1.35,  -0.55) | -0.30  (-0.70,  0.10) | -0.01^*^  (-0.01,  -0.003) | | -0.01^*^  (-0.01,  -0.01) |
| Adjusted R^2^ | Baseline | | | 0.76 | 0.74 | 0.365 | | 0.354 |
|  | Imaging | | | 0.76 | 0.74 | 0.0.369 | | 0.357 |
| Daytime dozing  (Yes/No, 95% CI) | | Baseline | | -2.10^*^  (-3.77,  -0.047) | -0.73  (-2.40,  0.94) | -0.02  (-0.04,  0.002) | | -0.01  (-0.03,  0.01) |
|  |  | Imaging | | -2.25^*^  (-3.84, -0.67) | -1.80  (-3.38,  -0.22) | -0.02  (-0.04,  -0.003) | | -0.01  (-0.03,  0.01) |
| Adjusted R^2^ | Baseline | | | 0.76 | 0.74 | 0.35 | | 0.34 |
|  | Imaging | | | 0.76 | 0.74 | 0.35 | | 0.34 |
| Snoring  (Yes/No, 95% CI) | | Baseline | | -0.17  (-1.26,  1.60) | 1.22  (-0.21,  2.64) | 0.01  (-0.01, 0.02) | | 0.005  (-0.01, 0.02) |
|  |  | Imaging | | 0.32  (-1.08,  1.72) | 0.54  (-0.86,  1.94) | 0.01  (-0.01,  0.03) | | 0.01  (-0.04, 0.03) |
| Adjusted R^2^ | Baseline | | | 0.76 | 0.74 | 0.34 | | 0.34 |
|  | Imaging | | | 0.76 | 0.74 | 0.34 | | 0.34 |
| Insomnia symptom  (Yes/No, 95% CI) | | Baseline | | 1.54  (0.07,  3.02) | 2.00^*^ (0.52,  3.47) | -0.004  (-0.02,  0.01) | | 0.01  (-0.01  0.03) |
|  |  | Imaging | | 2.10^*^  (0.52,  3.68) | 0.86  (0.71,  2.43 | 0.01  (-0.01,  0.03) | | 0.02  (0.003,  0.04) |
| Adjusted R^2^ | Baseline | | | 0.76 | 0.74 | 0.35 | | 0.34 |
|  | Imaging | | | 0.76 | 0.74 | 0.35 | 0.34 | |
| 95% CI, 95% confidence interval  *p-value <0.0125; p-value is considered significant after Bonferroni correction  † Sleep duration is centred on 7 hours of sleep per day  ‡ Normal sleep duration refers to 6-9 hours of sleep per day; Normal sleep duration is the reference group; Short sleep duration refers to less than 6 hours of sleep per day; Long sleep duration refers to more than 9 hours of sleep per day  The estimates are controlled for the following covariates: age, sex, education, body mass index, smoking, alcohol, physical activity, hypertension, and diabetes | | | | | | | | |

**Supplementary table 11.**

| **Supplementary table 11. Sensitivity analysis on the association between sleep characteristics and cognitive functions on participants with available depression measure (n=3,563).** | | | | | |
| --- | --- | --- | --- | --- | --- |
|  | | Dependent variable | | | |
| Independent variables | | **Visual memory**  **(Number of incorrect matches)** | **Reaction time**  **(Correct answer time in ms)** | **Numeric memory**  **(Number of correct answers)** | **Fluid intelligence**  **(Pooled score)** |
| Centred sleep duration†  (hours/day, 95% CI) | | -0.001  (-0.05, 0.05) | -0.03  (-3.14, 3.07) | -0.06^*^  (-0.10, -0.02) | 0.03  (-0.03, 0.10) |
| R^2^ adjusted | | 0.03 | 0.11 | 0.04 | 0.10 |
| Categorical sleep duration ‡ | Short | 0.28  (0.03, 0.53) | 1.47  (-10.60, 13.54) | 0.08  (-0.11, 0.28) | -0.42^*^  (-0.66, -0.17) |
|  | Long | 0.22  (-0.69, 0.26) | 20.06^*^  (5.72, 34.40) | -0.38^*^  (-0.56, -0.19) | -0.08  (-0.55, 0.39) |
| R^2^ adjusted | | 0.03 | 0.11 | 0.04 | 0.10 |
| Quadratic sleep duration | | 0.07^*^  (0.04, 0.11) | 0.79  (-2.72, 1.14) | -0.02  (-0.04, 0.01) | -0.08^*^  (-0.12, -0.04) |
| R^2^ adjusted | | 0.03 | 0.11 | 0.04 | 0.121 |
| Daytime dozing  (Yes/No, 95% CI) | | 0.13  (-0.03, 0.28) | 3.45  (-4.11, 11.02) | -0.01  (-0.11, 0.09) | -0.12  (-0.27, 0.04) |
| R^2^ adjusted | | 0.03 | 0.1 | 0.04 | 0.10 |
| Snoring  (Yes/No, 95% CI) | | 0.06  (-0.08, 0.19) | 0.51  (-6.14, 7.17) | -0.03  (-0.11, 0.06) | -0.03  (-0.16, 0.11) |
| R^2^ adjusted | | 0.03 | 0.11 | 0.04 | 0.10 |
| Insomnia symptom  (Yes/No, 95% CI) | | 0.04  (-0.11, 0.19) | 3.80  (-3.78, 11.38) | -0.01  (-0.11, 0.08) | -0.06  (-0.22, 0.09) |
| R^2^ adjusted | | 0.03 | 0.11 | 0.04 | 0.10 |
| 95% CI, 95% confidence interval  *p-value <0.0125; p-value is considered significant after Bonferroni correction  † Sleep duration is centred on 7 hours of sleep per day  ‡ Normal sleep duration refers to 6-9 hours of sleep per day; Normal sleep duration is the reference group; Short sleep duration refers to less than 6 hours of sleep per day; Long sleep duration refers to more than 9 hours of sleep per day  The estimates are controlled for the following covariates: age, sex, education, body mass index, smoking, alcohol, physical activity, hypertension, and diabetes | | | | | |

**Supplementary table 12.**

| Supplementary table 12. Characteristics of selected and not selected participants | | | | |
| --- | --- | --- | --- | --- |
|  | **Overall** | **Selected** | **Not selected** | **p-value** |
| n | 502506 | 29545 | 472961 |  |
| Demographics | | | | |
| Age at baseline visit (years)  mean (SD) | 56.53 (8.10) | 54.65 (7.44) | 56.65 (8.12) | <0.001 |
| Age at imaging visit (years)  Mean (SD) | 63.37 (7.55) | 62.98 (7.49) | 64.10 (7.62) | <0.001 |
| Sex (%)  Male | 229122 (45.6) | 13765 (46.6) | 215357 (45.5) | <0.001 |
| Education (%)  Higher | 161634 (39.5) | 13776 (49.1) | 147858 (38.8) | <0.001 |
| BMI at baseline visit (kg/m^2^)  mean (SD) | 27.43 (4.80) | 26.53 (4.20) | 27.49 (4.83) | <0.001 |
| BMI at imaging visit (kg/m^2^)  mean (SD) | 26.65 (4.51) | 26.50 (4.39) | 26.94 (4.72) | <0.001 |
| Smoking (%)  Never | 273550 (54.7) | 17943 (60.8) | 255607 (54.4) | <0.001 |
| Alcohol (%)  Never | 101773 (20.3) | 6661 (22.5) | 95112 (20.2) | <0.001 |
| Diabetes (%)  Yes | 26425 (5.3) | 738 (2.5) | 25687 (5.5) | <0.001 |
| Sleep measures | | | | |
| Sleep duration at baseline visit (hours/day)  mean (SD) | 7.10 (1.30) | 7.16 (0.99) | 7.09 (1.32) | <0.001 |
| Sleep duration at imaging visit (hours/day)  mean (SD) | 7.13 (1.13) | 7.14 (1.08) | 7.11 (1.21) | 0.01 |
| Snoring at baseline visit (%)  Yes | 173360 (37.2) | 10760 (36.4) | 162600 (37.3) | 0.002 |
| Snoring at imaging visit (%)  Yes | 16385 (36.3) | 10821 (36.6) | 5564 (35.8) | 0.078 |
| Insomnia symptoms at baseline visit (%)  Yes | 380226 (75.9) | 21331 (72.2) | 358895 (76.1) | <0.001 |
| Insomnia symptoms imaging visit (%)  Yes | 37953 (78.1) | 22950 (77.7) | 15003 (78.7) | 0.007 |
| Daytime dozing at baseline visit (%)  Yes | 120021 (24.1) | 5977 (20.2) | 114044 (24.3) | <0.001 |
| Daytime dozing at imaging visit (%)  Yes | 11537 (23.8) | 6781 (23.0) | 4756 (25.0) | <0.001 |
| Brain measures | | | | |
| Grey matter volume (ml)  mean (SD) | 665.23 (59.55) | 665.08 (59.53) | 665.62 (59.62) | 0.428 |
| White matter volume (ml)  mean (SD) | 477.99 (40.48) | 478.01 (40.47) | 477.96 (40.53) | 0.926 |
| Intracranial volume (ml)  mean (SD) | 1548.36 (152.21) | 1547.24 (152.01) | 1551.50 (152.76) | 0.014 |
| Left hippocampus volume (ml)  mean (SD) | 3.95  (0.41) | 3.96  (0.41) | 3.94  (0.41) | <0.001 |
| Right hippocampus volume (ml)  mean (SD) | 4.12  (0.43) | 4.13  (0.43) | 4.12  (0.42) | 0.007 |
| Cognitive measures | | | | |
| Visual memory at imaging visit  mean (SD) | 3.61  (2.91) | 3.57  (2.85) | 3.71  (3.01) | <0.001 |
| Reaction time at imaging visit  mean (SD) | 592.79 (109.00) | 590.65 (107.78) | 596.87 (111.19) | <0.001 |
| Numeric memory at imaging visit  mean (SD) | 6.70  (1.48) | 6.72  (1.46) | 6.67  (1.52) | 0.01 |
| Fluid intelligence at imaging visit  mean (SD) | 6.65  (2.07) | 6.67  (2.05) | 6.60  (2.10) | 0.002 |

# **2. Supplementary Methods**

This document contains information on methods used to collect data on sleep measures and cognitive functions from the UK Biobank participants.

**2.1. Sleep characteristics**

Measures of sleep characteristics were obtained in three instances: baseline, first follow-up and imaging visit (i.e., second follow-up).

Sleep measures are self-reported and include sleep duration, chronotype, difficulty getting up in the morning, napping, daytime dozing, snoring, and insomnia symptoms.

Sleep duration: was collected with the question “About how many hours of sleep do you get in every 24 hours? (Please, include naps)”.

Chronotype: Morning/evening person or chronotype was collected with the question “Do you consider yourself to be?”. The answers were reported in four factors: 1) more of a morning person, 2) more of a morning than evening person, 3) more of an evening person and 4) more of an evening than morning person.

Difficulty getting up in the morning: was determined with the question “On an average day, how easy do you find getting up in the morning?”. The answers were reported in three factors: 1) not at all easy, 2) not very easy, 3) fairly easy, and 4) very easy.

Nap: Frequency of nap during the day was collected with the question “Do you have a nap during the day?”. The answers were reported in three factors: 1) never/rarely, 2) sometimes and 3) usually.

Insomnia symptoms: was assessed with the question “Do you have trouble falling asleep at night or do you wake up in the middle of the night?”. The answers were reported in three factors: 1) never/rarely, 2) sometimes and 3) usually.

Snoring: was assessed with the question “Does your partner or a close relative or friend complain about your snoring?”. The answers were reported in two factors: 1) yes and 2) no.

Dozing: was assessed with the question “How likely are you to doze off or fall asleep during the daytime when you don’t mean to? (e.g. when working, reading or driving)”. The answers were reported in three factors: 1) never/rarely, 2) sometimes and 3) usually.

**2.2. Cognitive functions**

Cognitive functions including visual memory, reaction time, numeric memory, and fluid intelligence was assessed using touchscreen.

- - 1. **Visual memory**

The visual memory was assessed with 2 rounds of Pairs card game as shown in **Picture 1**. The participants were shown 3 pairs of cards that are then turned face-down. The participants were asked to match as many cards as possible by simply touching the cards and turning them face-up. This is repeated with six pairs of cards. The number of correct matches represented the visual memory performance.


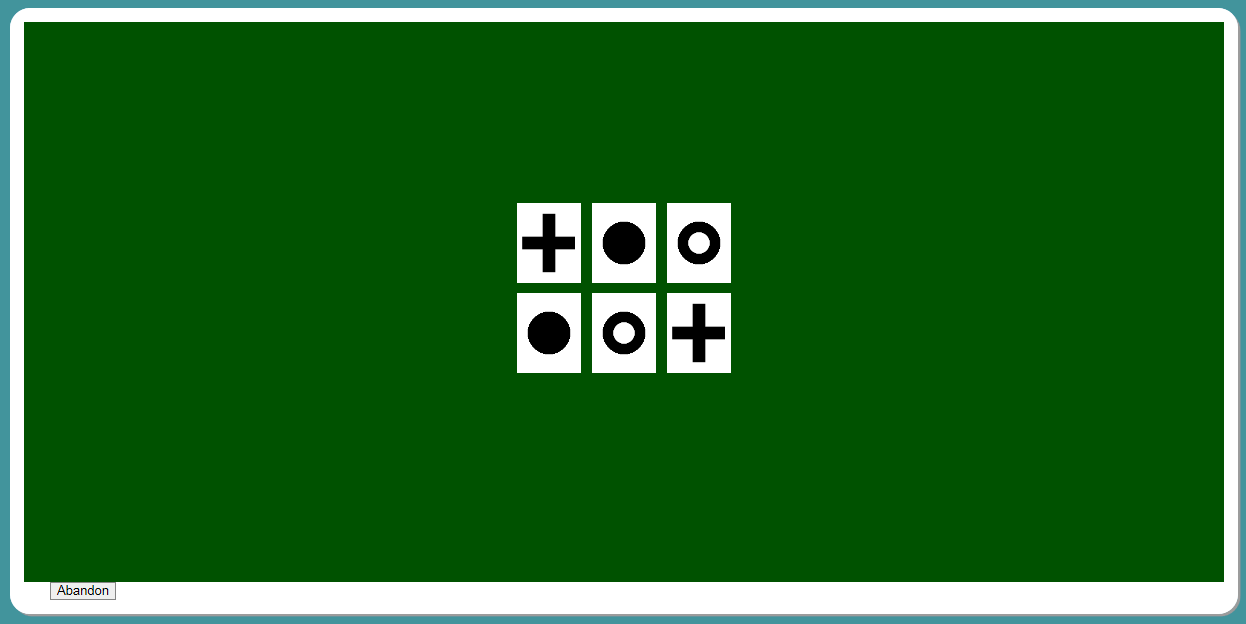


**Picture 1.** Visual memory assessment. Pairs card game. Three pairs of cards are shown for a period and turned face-down. Participants were asked to correctly match the pairs.

- - 1. **Reaction time**

The reaction time was assessed through 12 rounds of card-game called Snap as shown in **Picture 2.** Two cards were shown to the participant at a time. If both cards were the same, the participant was supposed to press the button as quickly as possible. The mean time to correctly identify the cards presented the reaction time.


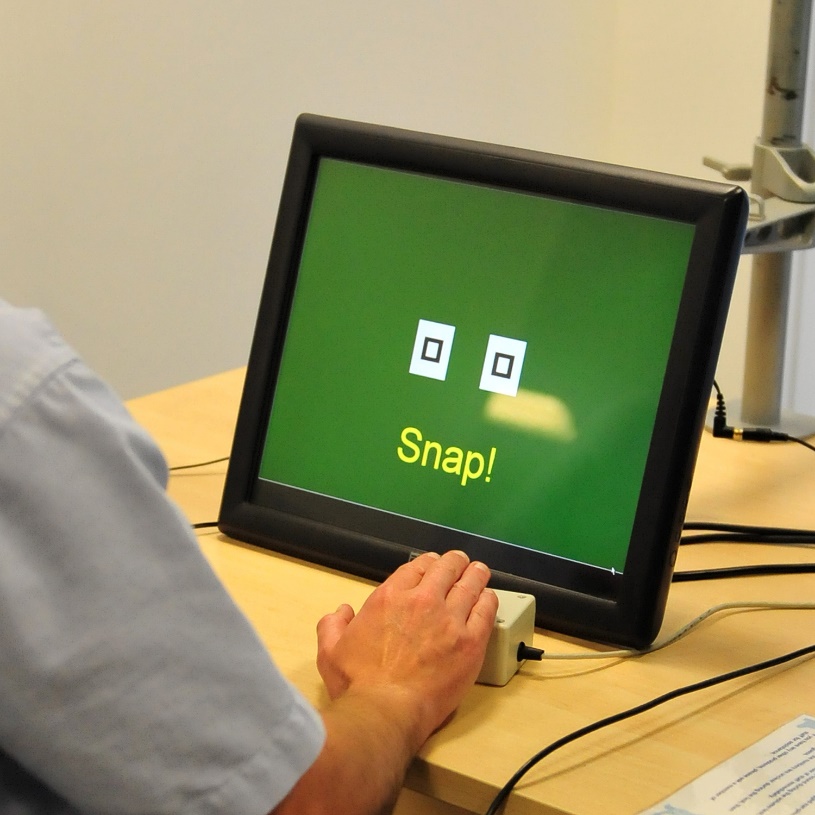


**Picture 2.** Reaction time assessment. Snap game. A participant is shown playing a snap game used to assess reaction time.

- - 1. **Numeric memory**

The numeric memory was assessed through memorization of 2-digit number which is displayed for 2000 ms as pictured in **Picture 3**. Then the numbers disappeared for 3000 ms. The participant was asked to remember and enter the number. The test starts with 2-digit number and each time the participant entered correct number the digit increased by 1 (up to maximum 12 digits). If the number was 2-digit, after 5 incorrect attempts the test ended. If the number was 3-digit or higher the test ended after 2 successive incorrect answers. The maximum digits remembered correctly represented the numeric memory performance.


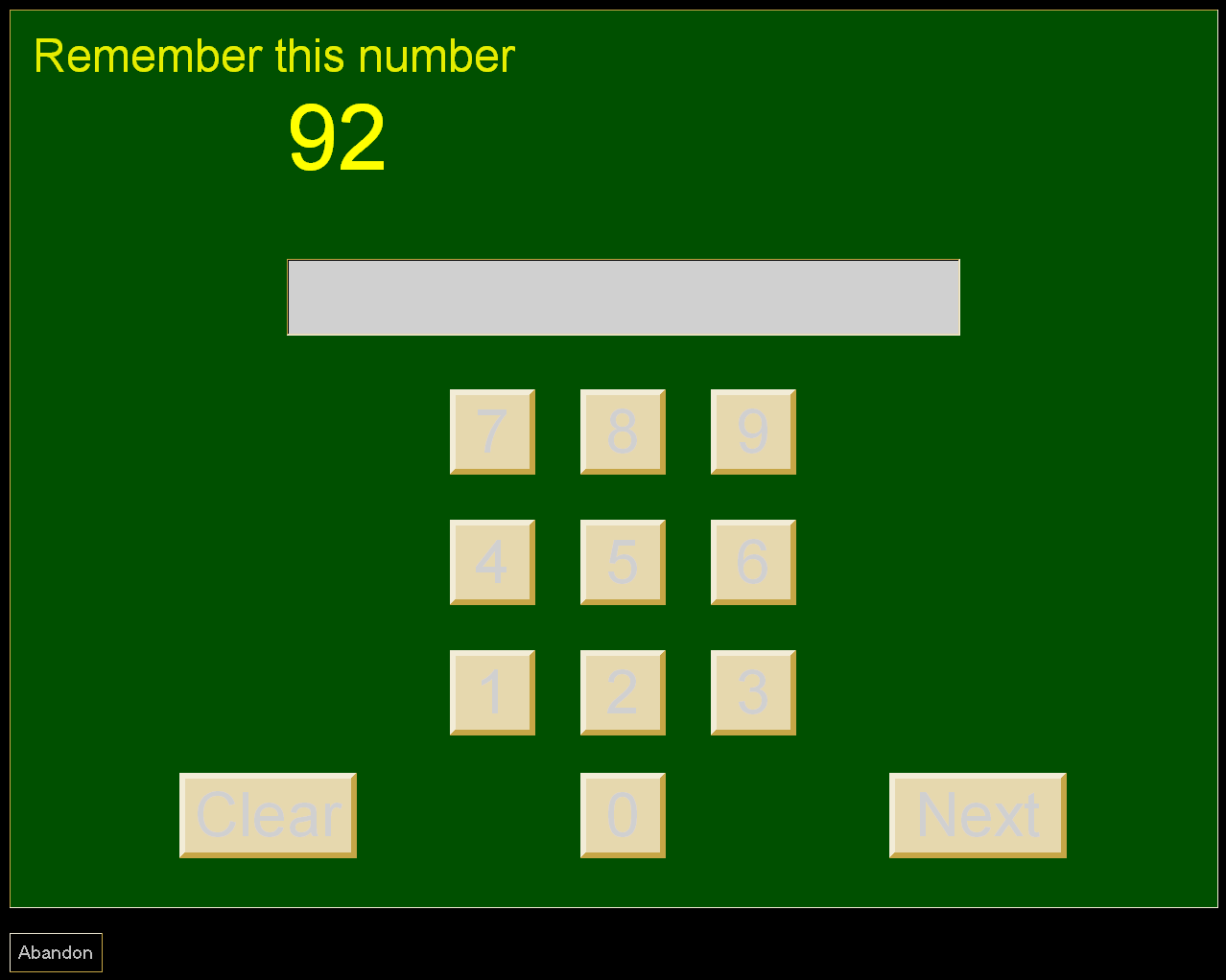


**Picture 3.** Numeric memory assessment. Number memorization. 2-digit number was displayed and disappeared for a period. Then the number was asked to be remembered and entered correctly.

- - 1. **Fluid intelligence**

Fluid intelligence was assessed through 13 questions designed to assess the capacity to solve problems that require logic and reasoning ability, independent of acquired knowledge. If the participant agreed to answer the questions a timer is started, and questions were displayed until 2 minutes had reached. The unweighted sum of 13 questions represented the fluid intelligence performance.
